# Interferon Regulatory Factor-1 (IRF-1) Shapes Both Innate and CD8^+^ T Cell Immune Responses against West Nile Virus Infection

**DOI:** 10.1371/journal.ppat.1002230

**Published:** 2011-09-01

**Authors:** James D. Brien, Stephane Daffis, Helen M. Lazear, Hyelim Cho, Mehul S. Suthar, Michael Gale, Michael S. Diamond

**Affiliations:** 1 Departments of Medicine, Washington University School of Medicine, St. Louis, Missouri, United States of America; 2 Molecular Microbiology, Washington University School of Medicine, St. Louis, Missouri, United States of America; 3 Department of Immunology, University of Washington, Seattle, Washington, United States of America; 4 Pathology and Immunology, Washington University School of Medicine, St. Louis, Missouri, United States of America; 5 Midwest Regional Center of Excellence for Biodefense and Emerging Infectious Diseases Research, Washington University School of Medicine, St. Louis, Missouri, United States of America; Mount Sinai School of Medicine, United States of America

## Abstract

Interferon regulatory factor (IRF)-1 is an immunomodulatory transcription factor that functions downstream of pathogen recognition receptor signaling and has been implicated as a regulator of type I interferon (IFN)-αβ expression and the immune response to virus infections. However, this role for IRF-1 remains controversial because altered type I IFN responses have not been systemically observed in *IRF-1* ^-/-^ mice. To evaluate the relationship of IRF-1 and immune regulation, we assessed West Nile virus (WNV) infectivity and the host response in *IRF-1* ^-/-^ cells and mice. *IRF-1* ^-/-^ mice were highly vulnerable to WNV infection with enhanced viral replication in peripheral tissues and rapid dissemination into the central nervous system. Ex vivo analysis revealed a cell-type specific antiviral role as *IRF-1* ^-/-^ macrophages supported enhanced WNV replication but infection was unaltered in *IRF-1* ^-/-^ fibroblasts. IRF-1 also had an independent and paradoxical effect on CD8^+^ T cell expansion. Although markedly fewer CD8^+^ T cells were observed in naïve animals as described previously, remarkably, *IRF-1* ^-/-^ mice rapidly expanded their pool of WNV-specific cytolytic CD8^+^ T cells. Adoptive transfer and in vitro proliferation experiments established both cell-intrinsic and cell-extrinsic effects of IRF-1 on the expansion of CD8^+^ T cells. Thus, IRF-1 restricts WNV infection by modulating the expression of innate antiviral effector molecules while shaping the antigen-specific CD8^+^ T cell response.

## Author Summary

Interferon regulatory factor (IRF)-1 is a transcription factor that has been implicated in immune regulation and induction of type I IFN gene expression. To better understand the contribution of IRF-1 to antiviral immunity, we infected cells and mice lacking IRF-1 with West Nile virus (WNV), an encephalitic flavivirus. *IRF-1* ^-/-^ mice were uniformly vulnerable to WNV infection with enhanced viral replication and rapid dissemination into the brain and spinal cord. Studies in cell culture revealed a cell-type specific antiviral role as *IRF-1* ^-/-^ macrophages but not fibroblasts supported enhanced WNV replication. IRF-1 also had an independent effect on CD8^+^ T cell responses. Although fewer CD8^+^ T cells were observed in naïve animals, WNV-specific CD8^+^ T cells rapidly expanded in *IRF-1* ^-/-^ mice and retained the capacity to clear infection. Collectively, our studies define independent roles for IRF-1 in restricting WNV pathogenesis and modulating the protective CD8^+^ T cell response.

## Introduction

The rapid triggering of an IFN-αβ response results in the early control of virus infection in mammalian cells. Detection of RNA viruses occurs through the recognition of specific sequence motifs, secondary structure, or modification of viral nucleic acids by pattern recognition receptors (PRR) in the cytosol (RIG-I and MDA5) and endosome (TLR3, TLR7, and TLR8) [1]. PRR binding of viral RNA signals constituent adaptor molecules (IPS-1, TRIF, and MyD88) to activate transcription factors and induce expression of IFN-αβ genes. A current paradigm for IFN production after RNA virus infection describes a positive feedback model that is modulated by the transcription factors interferon regulatory factors (IRF)-3 and IRF-7 [2], [3]. In the initial phase, viral nucleic acid sensing induces nuclear localization of IRF-3, which stimulates gene transcription and production of IFN-β and IFN-α4 by infected cells. In the second phase, these IFNs bind to the common IFN-αβ receptor in a paracrine and autocrine manner and signal through the JAK-STAT pathway resulting in the induced expression of hundreds of interferon stimulated genes (ISG) (e.g., PKR, RNAse L, viperin, ISG15, ISG54, ISG56, IFITM3, and ISG20), which limit viral replication through multiple mechanisms [4], [5], [6], [7], [8], [9]. IRF-7 is both an ISG and a transcriptional activator and participates in an IFN amplification loop by inducing IFN-β and many subtypes of IFN-α [3], [10].

West Nile virus (WNV) is a single-stranded positive polarity RNA virus in the *Flaviviridae* family that cycles in nature between birds and *Culex* mosquitoes. Humans, which are dead-end incidental hosts, can develop a febrile illness that progresses to flaccid paralysis, meningitis, or encephalitis [11]. WNV is emerging in the Western hemisphere as greater than 30,000 human cases of severe infection have been diagnosed in the United States since 1999, and millions have been infected and remain undiagnosed [12]. Experiments in mice have identified immune mechanisms of control with significant contributions from inflammatory cytokines, chemokines, complement, B CD4^+^, and CD8^+^ T cells (reviewed in [13], [14]). In particular, type I IFN (IFN-αβ) has an essential function in restricting cell and tissue tropism as *IFN-αβR* ^-/-^ or *IFN-β* ^-/-^ mice rapidly succumb to WNV infection [15], [16], [17].

Although IFN-β shapes a response that restricts WNV infection in many cells and tissues, its induction appears to differ in a cell type-dependent manner. In fibroblasts and neurons, IFN-β is triggered through an IPS-1-dependent and IRF-3 and IRF-7-dependent transcriptional signal [10], [18]. However, additional transcription factors contribute to the regulation of IFN-β as gene expression and protein production were preserved in *IRF-3* ^-/-^ x *IRF-7* ^-/-^ myeloid cells after WNV infection [19]. Based on these studies, we hypothesized that additional transcriptional factors could regulate IFN-β and/or ISG expression. In particular, the function of IRF-1 in the context of infection by WNV or other flaviviruses remained unknown. We viewed IRF-1 as a candidate transcription factor because it had been reported to contribute to IFN-β induction in some experimental systems. IRF-1 was initially identified as a Newcastle disease virus (NDV)-induced transcription factor that activated IFN-β gene transcription in cell culture [20], [21], [22]. Other experiments suggested that IRF-1 activation also regulated genes that directly limit replication of several viruses (encephalomyocarditis (EMCV), NDV, hepatitis C (HCV), yellow fever, Sindbis, and vesicular stomatitis (VSV) viruses) independently of IFN production [23], [24], [25]. In addition to these functions, IRF-1 transduces part of the IFN-γ signal (reviewed in [26]). Consistent with this, EMCV infection in *IRF-1* ^-/-^ fibroblasts was associated with a decrease in IFN-γ-stimulated genes [27], and IFN-γ-induced upregulation of cytokines and chemokines in macrophages (Mφ) required the transcriptional activity of IRF-1 [28], [29], [30], [31].

Studies with deficient mice have confirmed an important role for IRF-1 in controlling infection against EMCV or murine γ-herpesvirus 68 (γHV68) [27], [32]. However, *IRF-1* ^-/-^ mice did not show defects in the systemic type I IFN response in vivo after NDV infection [33] suggesting that it might regulate IFN genes in a pathogen and cell type-dependent manner. Independent of its possible effects on transducing IFN signals, IRF-1 has been defined as a tumor suppressor gene [34], [35], [36] that inhibits cell proliferation or enhances apoptosis in a manner dependent on its transcriptional activity (reviewed in [37], [38]). IRF-1 also regulates adaptive immune responses. Naïve *IRF-1* ^-/-^ mice exhibit blunted levels of CD8^+^ T cells in peripheral lymphoid organs in part, due to decreased class I MHC expression and impaired selection in the thymus [39], [40], [41]. Moreover, *IRF-1^-/-^* Mφ have decreased IL-12 production during bacterial and parasitic infection, whereas *IRF-1* ^-/-^ CD4^+^ T cells show reduced IL-12 receptor expression and are prone to T_H_2 skewing [42], [43], [44]. Nonetheless, because adoptive transfer of wild type peritoneal exudates cells restores induction of Th1 cells in *IRF-1* ^-/-^ mice, these defects are not entirely T cell intrinsic [45]. Finally, increased numbers of CD4^+^CD25^+^FoxP3^+^ regulatory T cells were reported in naïve *IRF-1* ^-/-^ mice [46].

Here, we evaluated the function of IRF-1 in the context of immunity to WNV infection. *IRF-1* ^-/-^ mice were vulnerable to WNV infection with enhanced viral replication in peripheral tissues and subsequent early dissemination to the brain and spinal cord. Despite a substantial baseline defect in the numbers of naïve CD8^+^ T cells after WNV infection, *IRF-1* ^-/-^ mice rapidly expanded antigen-specific IFN-γ-producing, granzyme B^+^ CD8^+^ T cells that were capable of killing targets and clearing virus from infected neurons. Although WNV-specific CD8^+^ T cells proliferated more rapidly in *IRF-1* ^-/-^ mice, they did not “catch up” quickly enough to mitigate the damage caused by early CNS dissemination that was due to impaired control of WNV in peripheral tissues.

## Results

### IRF-1 is required for control of lethal WNV infection

**Figure 1:**
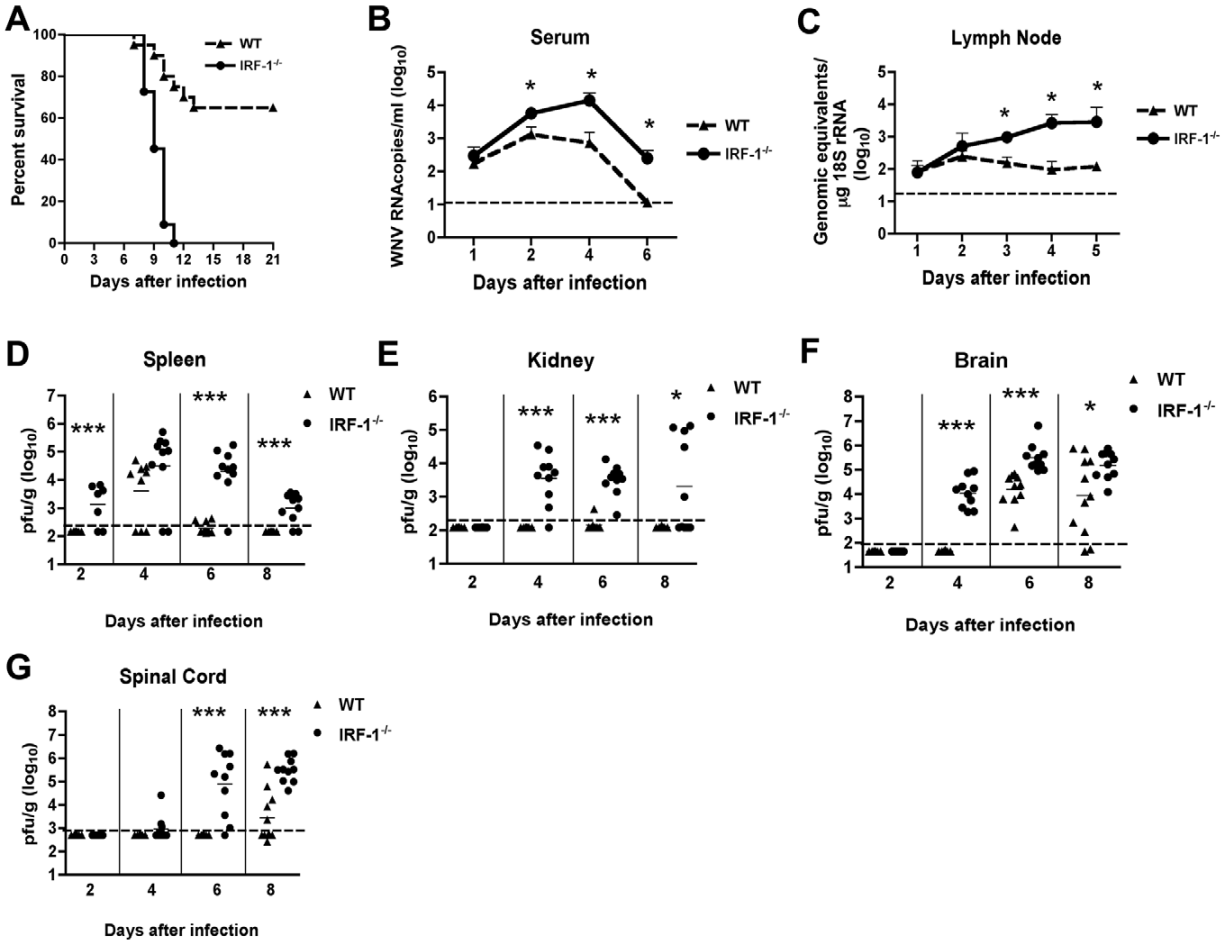
Survival and virologic analysis for wild type and *IRF-1* ^-/-^ C57BL/6 mice. (**A**) Eight to twelve week old mice were inoculated with 10^2^ PFU of WNV by footpad injection and followed for mortality for 21 days. Survival differences were statistically significant (n = 24, *IRF-1* ^-/-^; n = 20, wild type mice; P<0.0001). **B-G**. Viral burden in peripheral and CNS tissues after WNV infection. WNV RNA in (**B**) serum and (**C**) draining lymph node, and infectious virus in the (**D**) spleen, (**E**) kidney, (**F**) brain, and (**G**) spinal cord were determined from samples harvested on days 1,2, 3, 4, 5, 6, and 8 using qRT-PCR (**B** and **C**) or viral plaque assay (**D-G**) as indicated. Data are shown as viral RNA equivalents or PFU per gram of tissue for 7 to 10 mice per time point. For all viral load data, the solid line represents the median PFU per gram at the indicated time point, and the dotted line represents the limit of sensitivity of the assay. Error bars indicate the standard deviations (SD). Asterisks indicate values that are statistically significant (*, P<0.05; ***, P<0.0001) compared to wild type mice.

To understand the contribution of IRF-1 in controlling WNV infection in vivo, wild type and *IRF-1* ^-/-^ C57BL/6 mice were infected subcutaneously with 10^2^ PFU of a highly pathogenic WNV strain (New York 2000) and monitored for survival. *IRF-1* ^-/-^ mice were more vulnerable to WNV infection, with a 0% survival rate and a mean time to death of 9.5±1.1 compared to age-matched wild type mice, which had a 65% survival rate and a mean time to death of 10.7±1.6 (P<0.0001, **Fig. 1A** **)**.

### IRF-1^-/-^ mice exhibit enhanced WNV replication in peripheral and central nervous system tissues

To begin to define how an IRF-1 deficiency increased the susceptibility of mice to WNV infection, we measured viral burden by fluorogenic quantitative RT-PCR or viral plaque assay at days 1, 2, 4, 6 and 8 post infection in serum, peripheral organs (draining lymph nodes, spleen and kidney) and the CNS (brain and spinal cord).

#### (a) Peripheral tissues

Increased levels of viral RNA were observed in serum of *IRF-1* ^-/-^ mice at days 2, 4 and 6 when compared to wild type mice (4.3-, 21- and 22-fold higher, respectively, P<0.05) (**Fig 1B**). Similarly, in the draining lymph nodes, enhanced WNV replication also was observed at day 3, 4 and 5 after infection (**Fig 1C**, 6-, 27- and 23-fold reduction, respectively, P≤0.05). Thus, IRF-1 is required to control the early stages of WNV infection in vivo.

As seen previously [47], infectious WNV was not detected in the spleen of infected wild type animals until 4 days after infection. In contrast, 71% (5 of 7) of *IRF-1* ^-/-^ mice had measurable levels of WNV at day 2 (mean titer 10^3.1^ PFU/g, P<0.0001) (**Fig 1D**). Sustained viral replication was still observed at days 6 (9 of 10, mean titer 10^4.3^ PFU/g, P<0.0001) and 8 (8 of 10, 10^3.0^ PFU/g, P<0.0001), time points at which WNV was cleared from the spleen of wild type mice.

Normally, replication of WNV occurs to low, if at all detectable levels in the kidneys of adult wild type C57BL/6 mice [6]. In contrast, WNV infection of the kidney was observed in mice lacking components of the type I IFN signaling including the IFN- αβ receptor, IRF-3, or IRF-7 [10], [15], [47]. In the current study, WNV infection was detected in the kidneys of *IRF-1* ^-/-^ mice at days 4 (9 of 10, mean titer 10^3.5^ PFU/g, P<0.0001), 6 (10 of 10, mean titer 10^3.5^ PFU/g, P<0.0001) and 8 (5 of 10, mean titer 10^3.3^ PFU/g, P<0.05) (**Fig 1E**). Thus, IRF-1 restricts WNV tissue tropism and modulates infection in peripheral tissues.

#### (b) Central nervous system tissues

Dissemination of WNV to the central nervous system (CNS) of *IRF-1* ^-/-^ mice occurred more rapidly. Indeed, 100% of *IRF-1* ^-/-^ mice manifested WNV infection in the brain at day 4 (mean titer 10^3.7^ PFU/g, P<0.0001), a time at which no infectious WNV was detected in wild type animals (**Fig 1F**). Moreover, *IRF-1* ^-/-^ mice developed higher viral titers in the brain when compared to wild type animals on days 6 (mean titer 10^5.2^ PFU/g vs. 10^4.2^ PFU/g, P<0.0001) and 8 (mean titer 10^5.2^ PFU/g vs. 10^3.9^ PFU/g, P<0.05). Similarly, early infection of the spinal cord was observed in *IRF-1* ^-/-^ mice with 20% (2 of 10) and 90% (9 of 10) having detectable viral loads at day 4 (10^3.1^ PFU/g) and at day 6 (10^4.9^ PFU/g, P<0.0001), respectively (**Fig 1G**), whereas infection in wild type mice was not observed at these times. Higher titers in *IRF-1* ^-/-^ mice were also observed at day 8 (mean titer 10^5.5^ PFU/g vs. 10^3.4^ PFU/g, P<0.0001). Collectively, these data show that an absence of IRF-1 resulted in early spread and sustained WNV replication in CNS tissues.

### An absence of IRF-1 does not alter type I IFN levels in the draining lymph node or serum

**Figure 2:**
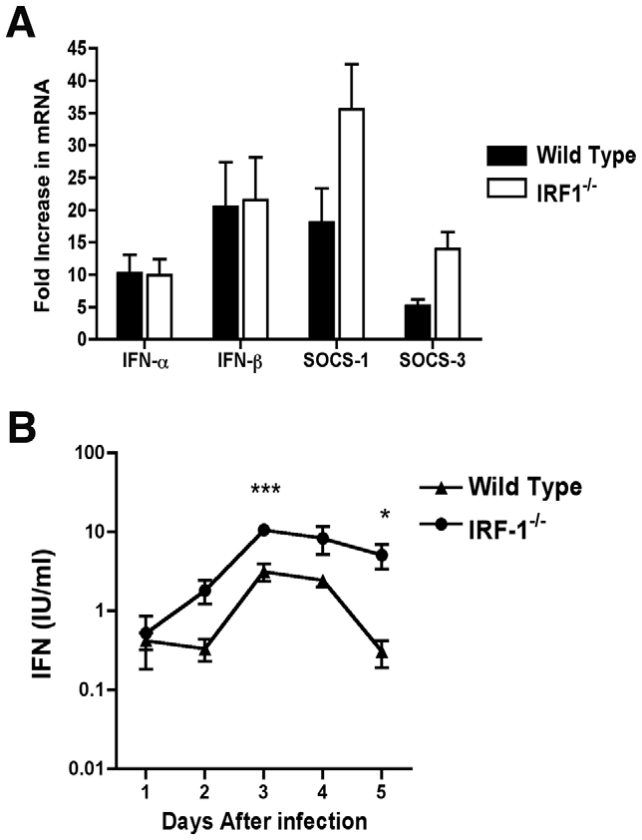
IFN-dependent responses in lymph node and serum of wild type and *IRF-1* ^-/-^ mice infected with WNV. **A**. Mice were inoculated with 10^2^ PFU of WNV by footpad injection and sacrificed at 2 days post infection. After harvesting of the draining inguinal lymph node, IFN-α, IFN-β, SOCS-1, and SOCS-3 mRNA levels were measured by quantitative RT-PCR and compared to mock-infected animals. **B**. Type I IFN levels were determined from serum collected on days 1 through 5 after WNV infection of wild type or *IRF-1* ^-/-^ mice by an EMCV bioassay in L929 cells. Data reflect averages of serum samples from 3 to 7 mice per time point and the data are expressed as international units (IU) of type I IFN per ml. The specificity of the assay was confirmed with an anti-IFN-αβR neutralizing antibody (data not shown). Asterisks indicate values that are statistically significant (***, P<0.001; *, P<0.05).

Because of the early viral phenotype in peripheral tissues, and prior results with other viruses implicating IRF-1 as an activator of IFN-αβ gene transcription in cell culture [20], [21], [22], we hypothesized that IRF-1 might contribute to induction of type I IFN responses locally in specific tissues or compartments. To test this, we measured levels of IFN-α and β mRNAs in the draining lymph nodes of WNV-infected mice at day 2 after infection. In wild type and *IRF-1* ^-/-^ mice, the induction of the IFN-α/β genes was comparable as both showed increased levels of IFN-α and β mRNA after WNV infection (∼10 to 20 fold increase for IFN-α and IFN-β) (**Fig 2A**). We also observed no statistically significant difference in induction of SOCS-1 and SOCS-3 in the draining lymph node, genes that are induced by IFN-γ [48].

Although IRF-3 and IRF-7 jointly regulate the induction of systemic IFN-αβ after WNV infection, their combined deficiency does not abrogate IFN accumulation in serum [19]. We therefore hypothesized that IRF-1 might contribute to the systemic levels of IFN-αβ in circulation after infection. Mice were infected with WNV and the presence of biologically active IFN in serum was monitored using a previously validated EMCV-L929 cell bioassay [47], [49]. Type I IFN activity in the serum of infected wild type mice peaked at day 3 and then decreased by day 5 (**Fig 2B**). IFN activity in the WNV-infected *IRF-1* ^-/-^ mice was equivalent or greater than that observed in wild type mice. For example, 5 to 10-fold higher levels of type I IFN activity were observed in *IRF-1* ^-/-^ mice at day 3 after infection (P<0.001). Thus, a deficiency of IRF-1 in vivo did not diminish early type I IFN levels in lymphoid tissues or circulation after WNV infection; these results are consistent with that observed previously after NDV infection [33].

### IRF-1 restricts WNV replication in macrophages but does not affect IFN-αβ induction

**Figure 3:**
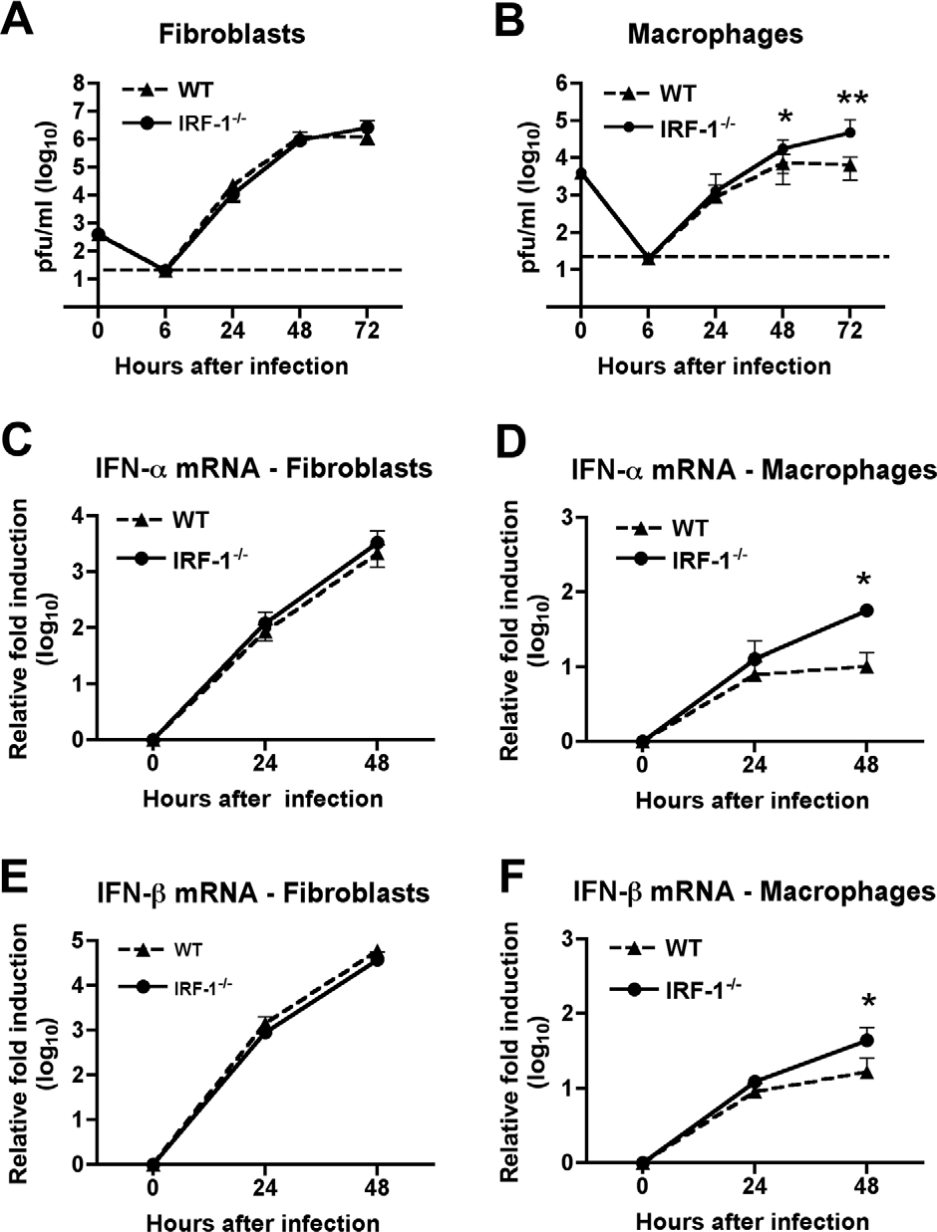
IRF-1 modulates WNV infection in primary Mφ but not in MEF. **A-B**. MEF (**A**) or Mφ (**B**) generated from wild type or *IRF-1* ^-/-^ mice were infected, and virus production was evaluated at the indicated times post infection by plaque assay. Values are an average of quadruplicate samples generated from at least three independent experiments, error bars represent the SD, and asterisks indicate differences that are statistically significant relative to wild type mice (*, P<0.05; **, P<0.005). **C-F**. The induction of IFN-α (**C-D**) or IFN-β (**E-F**) mRNA in WNV-infected MEF (**C, E**) or Mφ (**D, F**) was analyzed by qRT-PCR. The data are the average of at least three independent experiments performed in triplicate, error bars represent the SD, and asterisks indicate differences that are statistically significant relative to wild type mice (*, P<0.05).

To better understand the viral replication phenotype of *IRF-1* ^-/-^ mice, we investigated how an absence of IRF-1 affects WNV infection of primary mouse embryonic fibroblasts (MEF) and Mφ. While Mφ are physiologic targets of WNV infection in vivo [6], [50], [51], we used MEFs as a comparison cell type because historically they have been used to study the significance of IRF in regulating cellular antiviral immune responses [3], [19], [52]. Multi-step viral growth curves performed in wild type and *IRF-1* ^-/-^ MEF showed no significant differences in WNV replication at any of the time points tested (P > 0.2) (**Fig 3A**). However, increased replication was observed at 48 and 72 hours after infection in *IRF-1* ^-/-^ Mφ when compared to wild type cells (**Fig 3B**, 2.5-fold, P = 0.02 and 7.4-fold, P = 0.001, respectively). Earlier studies showed that a defect in type I IFN signaling enhanced WNV infection of primary Mφ [15] while recent work indicated that IRF-3 and IRF-7 only partially regulated IFN-β induction after WNV infection in Mφ [19]. To define a possible role for IRF-1 in modulating IFN gene induction, cells were infected with WNV and levels of IFN-α and β mRNA were measured by qRT-PCR. Notably, levels of IFN-α and IFN-β were significantly higher at 48 hours in *IRF-1* ^-/-^ Mφ (**Fig 3D and F**); the increased levels were likely a consequence of greater viral replication, analogous to that seen in *MyD88* ^-/-^ Mφ [53]. Consistent with this, IFN-αβ gene induction was not altered in *IRF-1* ^-/-^ MEF (**Fig 3C and E**). Thus, a deficiency in IRF-1 enhanced viral growth in Mφ yet this was not associated with a defect in the type I IFN response.

### IRF-1 mediates IFN-γ but not IFN-β-dependent antiviral effects in macrophages

**Figure 4:**
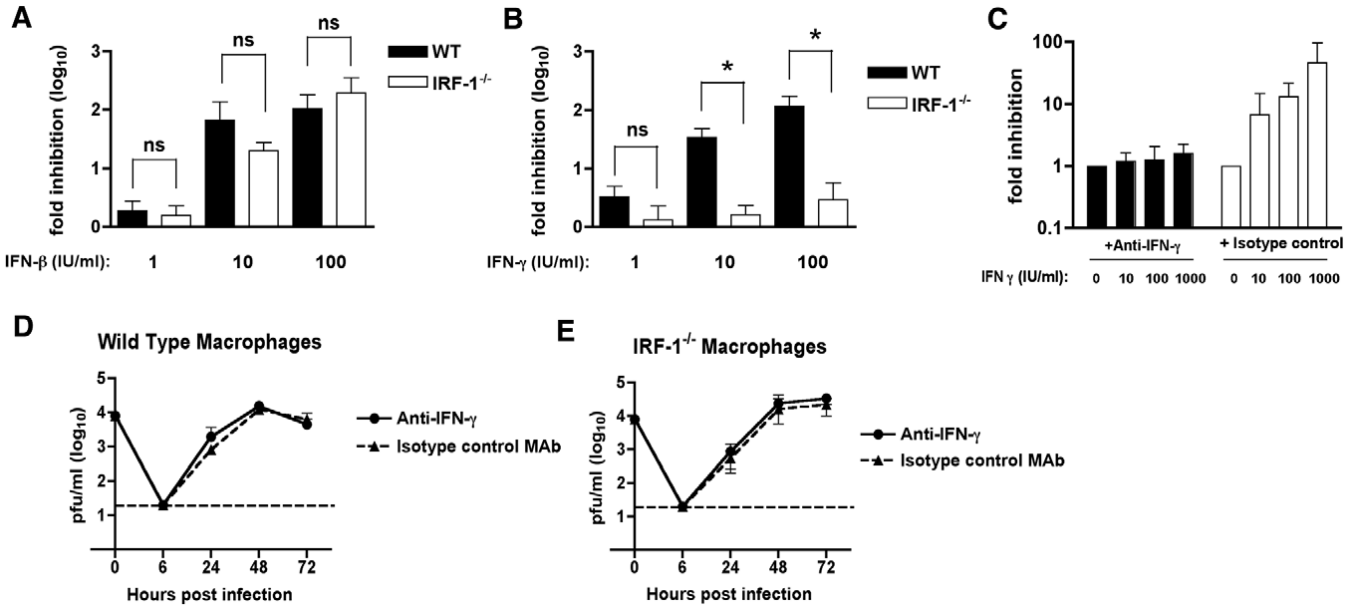
The relationship between IFN-γ and IRF-1 in Mφ. **A-B**.The antiviral effects of exogenous IFN-β or IFN-γ in Mφ. Wild type or *IRF-1* ^-/-^ Mφ were pretreated overnight with the indicated amount of (**A**) IFN-β (1 to 100 IU/ml) or (**B**) IFN-γ (1 to 100 IU/ml). Subsequently, cells were infected with WNV at an MOI of 0.01 and one-day later supernatants were harvested and titrated for viral infection. The data are expressed as the log_10_ fold inhibition relative to the no IFN treatment control. Asterisks indicate differences that are statistically significant (*, P<0.05). **C-E**. The antiviral effect of IRF-1 in macrophages is independent of IFN-γ expression. **C**. The H22 MAb efficiently neutralizes the antiviral effect of exogenous IFN-γ treatment. Mφ were pretreated with the indicated concentrations of IFN-γ in the presence of H22 anti-IFN-γ or an isotype control MAb. Subsequently, cells were infected with WNV at an MOI of 0.01 and one-day later supernatants were harvested and titrated for viral infection. The data are expressed as the log_10_ fold inhibition relative to the no IFN treatment control. **(D)** Wild type or (**E**) *IRF-1* ^-/-^ Mφ were infected at an MOI of 0.01 in the presence of H22 anti-IFN-γ or an isotype control MAb. Virus production was evaluated at the indicated times by plaque assay. Values are an average of triplicate samples generated from three independent experiments, and error bars represent the SD. Differences were not statistically significant.

Since previous studies have defined a role of IRF-1 in mediating the antiviral effects of IFN-γ signaling [54], we evaluated the contribution of IRF-1 to the antiviral effects of either IFN-β or IFN-γ in Mφ after WNV infection. We pretreated wild type and *IRF-1* ^-/-^ Mφ with increasing concentrations of either IFN-β or IFN-γ, infected with WNV, and measured viral burden. Pretreatment with increasing amounts of IFN-β inhibited WNV replication in both wild type and *IRF-1* ^-/-^ Mφ (2-, 66- and 104-fold inhibition versus 1.6-, 20- and 193-fold inhibition, respectively, P>0.3 at 1, 10 and 100 IU/ml) (**Fig 4A**). Whereas pretreatment with IFN-γ significantly decreased viral infection in wild type Mφ, it had virtually no inhibitory effect in *IRF-1* ^-/-^ cells (3.3-, 34- and 116-fold inhibition in wild type cells versus 1.3-, 1.6- and 2.9-fold inhibition in *IRF-1* ^-/-^ cells, P<0.05 at 1, 10 and 100 IU/ml) (**Fig 4B**). Thus, in Mφ, IRF-1 is required to mediate the inhibitory effect of IFN-γ but dispensable for the antiviral activity of IFN-β against WNV infection.

To determine whether the increased WNV replication in *IRF-1* ^-/-^ Mφ was associated with a paracrine antiviral effect of IFN-γ, we performed depletion experiments with a neutralizing anti-mouse IFN-γ monoclonal antibody (MAb H22) [55]. To confirm the neutralizing capacity of the H22 MAb, wild type Mφ were pretreated with increasing amounts of IFN-γ that was pre-mixed with either H22 or an isotype control MAb, and then infected with WNV; at 48 hours, viral burden was quantified by plaque assay. Pre-incubation of IFN-γ with the isotype control MAb resulted in a dose-dependent inhibition in WNV infection (**Fig 4C**). Since these controls confirmed that H22 MAb neutralized the IFN-γ-dependent antiviral activity against WNV, we performed multi-step viral growth in wild type and *IRF-1* ^-/-^ Mφ in the presence of H22 or the isotype control MAb, each in the absence of exogenous IFN-γ. Notably, WNV replication in the presence of the isotype control MAb gradually increased in both wild type and *IRF-1* ^-/-^ Mφ (**Fig 4D and 4E**) and exhibited growth kinetics virtually identical to that observed in the absence of MAb (see **Fig 3B**). Similar results were obtained on WNV growth in the presence of H22. These results imply that WNV-infected Mφ did not produce sufficient IFN-γ to explain the difference in viral growth between wild type and *IRF-1* ^-/-^ cells. Thus, although IRF-1 is essential for mediating the antiviral activity of IFN-γ against WNV in Mφ, the cell-intrinsic difference in replication in *IRF-1* ^-/-^ cells must occur independently of the IFN-γ response.

### WNV-specific B cell responses remain intact in IRF-1^-/-^ mice

**Figure 5:**
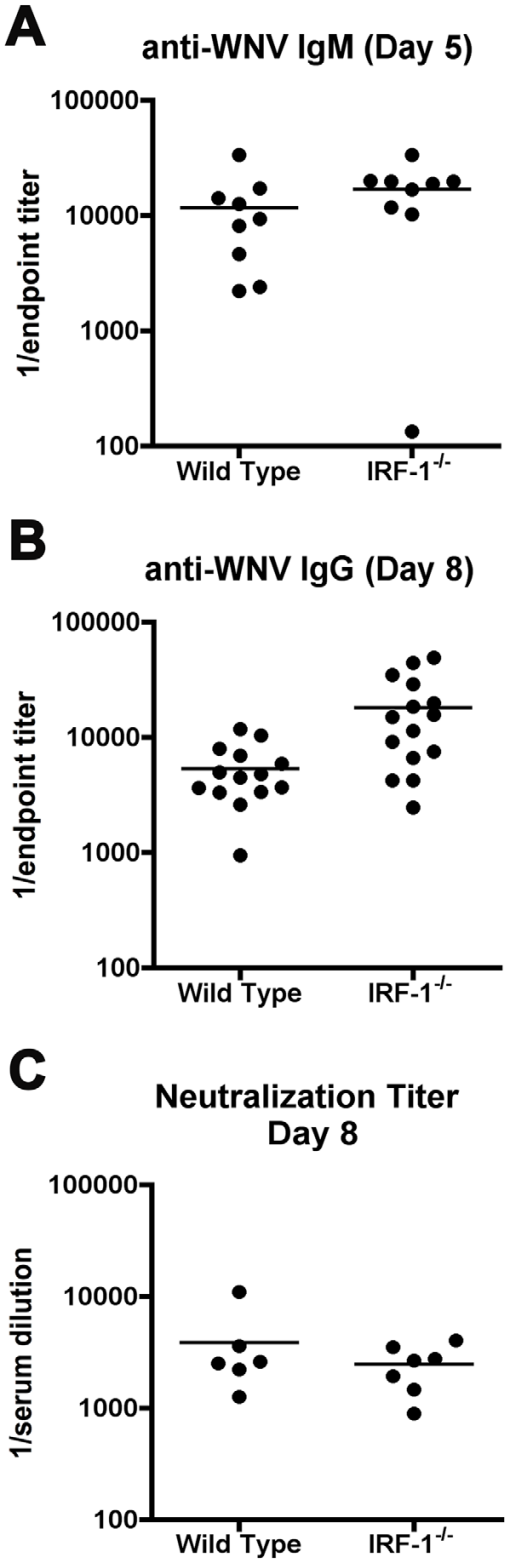
Antibody responses in *IRF-1* ^-/-^ mice remain intact after WNV infection. **A-B.** Wild type and *IRF-1* ^-/-^ mice were inoculated with 10^2^ PFU of WNV-NY by footpad injection and serum samples collected on days 5 and 8 after subcutaneous WNV infection were assayed by ELISA for WNV-E specific (**A**) IgM and (**B**) IgG. Titers are expressed as a scatter plot of the reciprocal serum dilution that was three standard deviations above background (endpoint titer). All IgM and IgG titers were not statistically significant from wild type mice (P > 0.1). **C**. Neutralizing antibody titers in wild type and *IRF-1* ^-/-^ mice from serum collected at day 8 after infection. Filled circles reflect samples from individual mice and reflect the serum dilution that inhibits 50% of infection of Vero cells as judged by a focus reduction neutralization assay. No statistical difference (P > 0.3) was observed.

Innate immune signaling can modulate the induction and quality of antigen-specific antibody responses after viral infection [18], [56]. As a depressed antiviral antibody response can promote increased viremia and early dissemination of WNV [57], we evaluated whether a deficiency of IRF-1 modulated humoral immune responses. Notably, similar or higher levels of WNV-specific IgM and IgG were detected in *IRF-1* ^-/-^ mice at days 5 and 8 after infection, and no difference in neutralization titer was observed (**Fig 5**). Thus, the virologic phenotype observed in *IRF-1* ^-/-^ mice likely was not due to a primary defect in B cell function.

### WNV-specific CD8^+^ T cell responses are hyperactive in IRF-1^-/-^ mice

**Figure 6:**
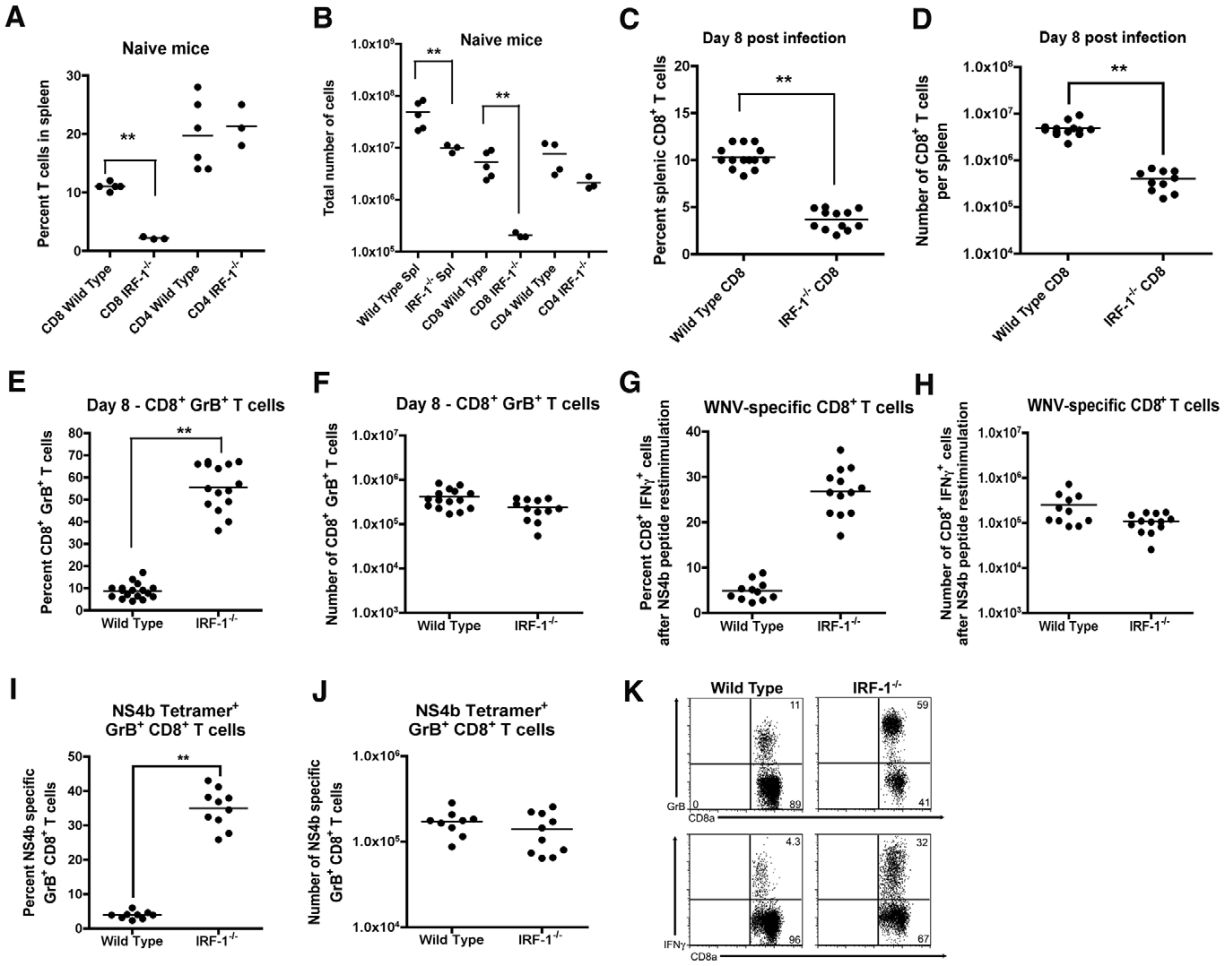
Splenic T cell responses in *IRF-1* ^-/-^ mice. **A-B**. The total percentage (**A**) and number (**B**) of CD4^+^ and CD8^+^ T cells were obtained from naïve mice and determined by flow cytometry. A scatterplot of the data is shown and was generated from three to six wild type or *IRF-1* ^-/-^ mice. The bar indicates average values and asterisks denote differences that are statistically significant (P<0.05). **C-H** Wild type and *IRF-1* ^-/-^ mice were inoculated with 10^2^ PFU of WNV by footpad injection and spleens were harvested on day 8. **C-D**. The total percentage (**C**) and number (**D**) of CD8^+^ T cells were determined by flow cytometry. A scatterplot of the data is shown and was generated from 12 to 14 wild type or *IRF-1* ^-/-^ mice. The bar indicates average values and asterisks denote differences that are statistically significant (P<0.05). **E-F**. The total percentage (**E**) and number (**F**) of splenic CD8^+^ T cells expressing GrB. Cells were harvested directly from dissected spleens and analyzed by flow cytometry (**K**, *top dot plots*). A scatterplot of the data is shown and was generated from 12 to 14 wild type or *IRF-1* ^-/-^ mice. The bar indicates average values and asterisks denote differences that are statistically significant (P<0.05). **G-H**. Splenocytes were stimulated ex vivo with D^b^-restricted NS4b peptide, stained for CD3 and CD8b, and intracellular IFN-γ, and analyzed by flow cytometry. The primary data is shown as dot plots of gated CD3^+^CD8^+^ cells (**K,** *bottom dot plot*) and (**G**) a summary of the percentage and (**H**) total number of CD3^+^CD8^+^ T cells positive for intracellular IFN-γ after peptide restimulation. A scatterplot of the data is shown and was generated from 12 to 14 wild type or *IRF-1* ^-/-^ mice. Note, the data in panel G is presented with the background IFN-γ staining subtracted: wild type CD8^+^ IFN-γ^+^ background of 0.1% (*n* = 14 mice) and IRF-1^-/-^ CD8^+^ IFN-γ^+^ background of 0.2% (*n* = 12). The bar indicates average values and asterisks denote differences that are statistically significant (P<0.05). **I-J**. The total percentage (**I**) and number (**J**) of splenic CD8^+^ T cells co-expressing GrB and D^b^-NS4b peptide as judged by tetramer staining and flow cytometry. A scatterplot of the data from three independent experiments is shown and was generated from 9 to 10 wild type or *IRF-1* ^-/-^ mice. The bar indicates average values and asterisks denote differences that are statistically significant (P<0.05).

Naïve *IRF-1* ^-/-^ mice have quantitative defects in the number of CD8^+^ T cells in peripheral lymphoid organs because of impaired negative and positive selection in the thymus [39], [40], [41]. Moreover, *IRF-1* ^-/-^ mice also have defects in T_H_1 responses due to altered production of IL-12 by Mφ and hypo-responsiveness of T cells to the effects of IL-12 during bacterial and parasite infections [42], [43], [44]. Because WNV is controlled in part, by cytolytic and IFN-γ secreting CD8^+^ T cells that clear infection from peripheral and CNS tissues [58], [59], [60], [61], [62], we evaluated whether the severe clinical phenotype after infection in *IRF-1* ^-/-^ mice was explained in part, by a failure of antigen-specific cells to expand and migrate to infected tissues. Initial studies confirmed a reduced percentage (∼5-fold, P<0.001) and number (∼25-fold, P<0.001) of CD8^+^ T cells in the spleens of naïve *IRF-1* ^-/-^ mice (**Fig 6A and B**). In comparison, the percentage and absolute number of CD4^+^ T cells were largely intact in naive *IRF-1* ^-/-^ mice. However, within eight days of WNV infection, the total CD8^+^ T cell population expanded in *IRF-1* ^-/-^ mice, such that there was a smaller difference in percentage (∼3-fold) and number (∼10-fold) compared to wild type mice (**Fig 6C and D**). Remarkably, ∼60% of splenic CD8^+^ T cells taken directly from WNV-infected *IRF-1* ^-/-^ mice expressed high levels of granzyme B (GrB), establishing the presence of a large pool of potentially cytolytic CD8^+^ T cells (**Fig 6E, F, and K**); the majority of these GrB^+^ CD8^+^ T cells were specific for one D^b^-restricted immunodominant epitope on the WNV NS4b protein (**Fig 6I and J** **, and Fig S1**). In comparison, ∼0.2% of CD8^+^ T cells expressed GrB in naïve *IRF-1* ^-/-^ mice (data not shown). Consistent with this, a significantly higher percentage (∼30% compared to ∼5% for wild type, P = 0.001) of *IRF-1* ^-/-^ CD8^+^ T cells produced IFN-γ in response to WNV NS4b peptide restimulation ex vivo (**Fig 6G, H, and K**). This result was not specific to the NS4b epitope as similar results were observed after re-stimulation with an E protein-derived peptide (E_771_: IALTFLAV) that comprises a subdominant K^b^-restricted T cell epitope (data not shown). In comparison, neither wild type nor *IRF-1* ^-/-^ CD8^+^ T cells from WNV-infected mice produced IFN-γ in the absence NS4b or E peptide restimulation (0.1 to 0.2% IFN-γ^+^ CD8^+^ positive cells from wild type and *IRF-1* ^-/-^ mice, respectively). Overall, in the context of WNV infection, *IRF-1* ^-/-^ mice paradoxically had more robust antigen-specific T cell responses.

### Mechanism of selective expansion of IRF-1^-/-^ CD8^+^ T cells

**Figure 7:**
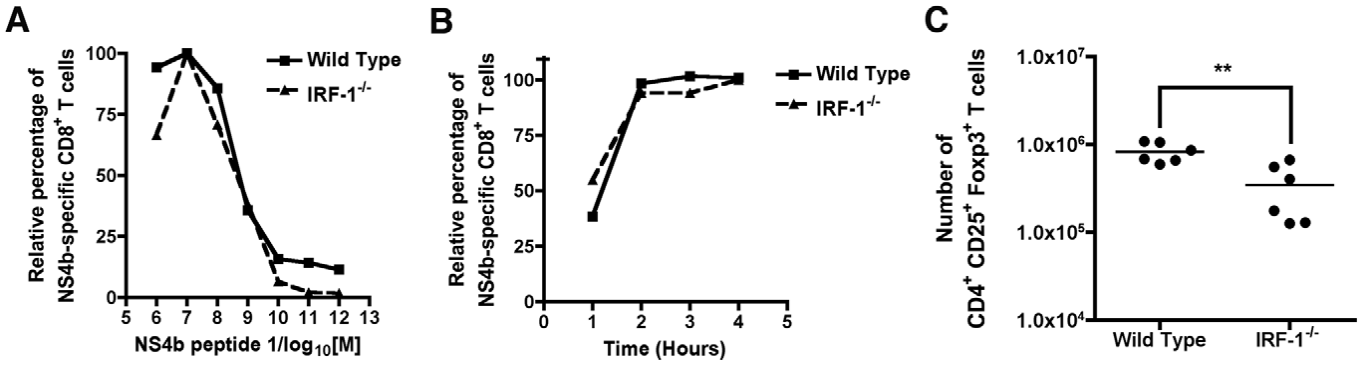
Mechanism of selective expansion of WNV-specific CD8^+^ T cells in *IRF-1* ^-/-^ mice. **A**. Assessment of CD8^+^ T cell avidity. Ex vivo restimulation of CD8^+^ T cells from wild type or *IRF-1* ^-/-^ mice was performed over the indicated range of NS4b peptide concentrations. Cells were stained for CD3, CD8b, and intracellular IFN-γ, and analyzed by flow cytometry. The data are normalized relative to the peak percentage of cells expressing IFN-γ (for wild type (∼5%) or *IRF-1* ^-/-^ (∼30%)) after incubation with NS4b peptide (10^-7^ M). Data were generated from two independent experiments with 6 to 8 wild type or *IRF-1* ^-/-^ mice in total. **B**. Kinetics of CD8^+^ T cell induction. Wild type or *IRF-1* ^-/-^ CD8^+^ T cells from WNV-infected mice were harvested at day 8 and restimulated ex vivo with NS4b peptide (10^-7^ M) for the indicated times. Cells were stained for CD3, CD8b, and intracellular IFN-γ, and analyzed by flow cytometry. The data are normalized relative to the peak percentage of cells expressing IFN-γ (for wild type (∼5%) or *IRF-1* ^-/-^ (30%) after incubation with NS4b peptide (6 hour time point). Data were generated two independent experiments with 6 to 8 wild type or *IRF-1* ^-/-^ mice in total. **C**. Treg analysis in WNV-infected wild type or *IRF-1* ^-/-^ mice. At day 8, spleens were harvested and analyzed for the number of Treg using antibodies to CD4, CD25, and FoxP3. Data are displayed as a scatterplot and reflect six individual wild type or *IRF-1* ^-/-^ mice from two independent experiments. Asterisks indicate values that are statistically different.

The rapid and antigen-specific expansion of CD8^+^ T cells in *IRF-1* ^-/-^ mice after WNV infection was surprising, given the previously described defects in negative and positive T cell selection in the thymus and IL-12 responsiveness in the periphery. We hypothesized that one of several mechanisms could explain this phenomenon: (a) WNV-specific *IRF-1* ^-/-^ CD8^+^ T cells were inherently of higher affinity because they had been selected on *IRF-1* ^-/-^ stromal cells expressing lower levels of class I MHC molecules [39]; (b) IRF-1 directly or indirectly modulated the numbers of CD4^+^CD25^+^FoxP3^+^ regulatory T cells (Treg) after WNV infection. A decrease in Treg numbers was recently shown to cause an increase in numbers of WNV-specific CD8^+^ T cells [63]; or (c) IRF-1 regulated the threshold for activation-induced proliferation after antigen recognition, consistent with its tumor suppressor properties. We tested these three hypotheses in the following ways. To evaluate whether WNV-specific *IRF-1* ^-/-^ CD8^+^ T cells were of higher affinity than wild type CD8^+^ T cells, ex vivo restimulation was performed over a wide range of NS4b peptide concentrations. Notably, no difference was observed in the concentration of peptide or the time required for activation of CD8^+^ T cells and expression of IFN-γ (**Fig 7A and B**). Moreover, we observed a slightly (2.4-fold, P = 0.004) decreased number of CD4^+^CD25^+^FoxP3^+^ Tregs in the spleens of WNV-infected *IRF-1* ^-/-^ mice, which could contribute to the skewing of the CD8^+^ T cell response (**Fig 7C**). However, this reduction is an unlikely explanation because the CD8^+^ T cell response disparity in *IRF-1* ^-/-^ mice was far greater than that observed in *FoxP3* ^-/-^ mice, which entirely lack Tregs [**63**].

### Cell-intrinsic and –extrinsic defects of IRF-1 alter CD8^+^ T cell expansion

**Figure 8:**
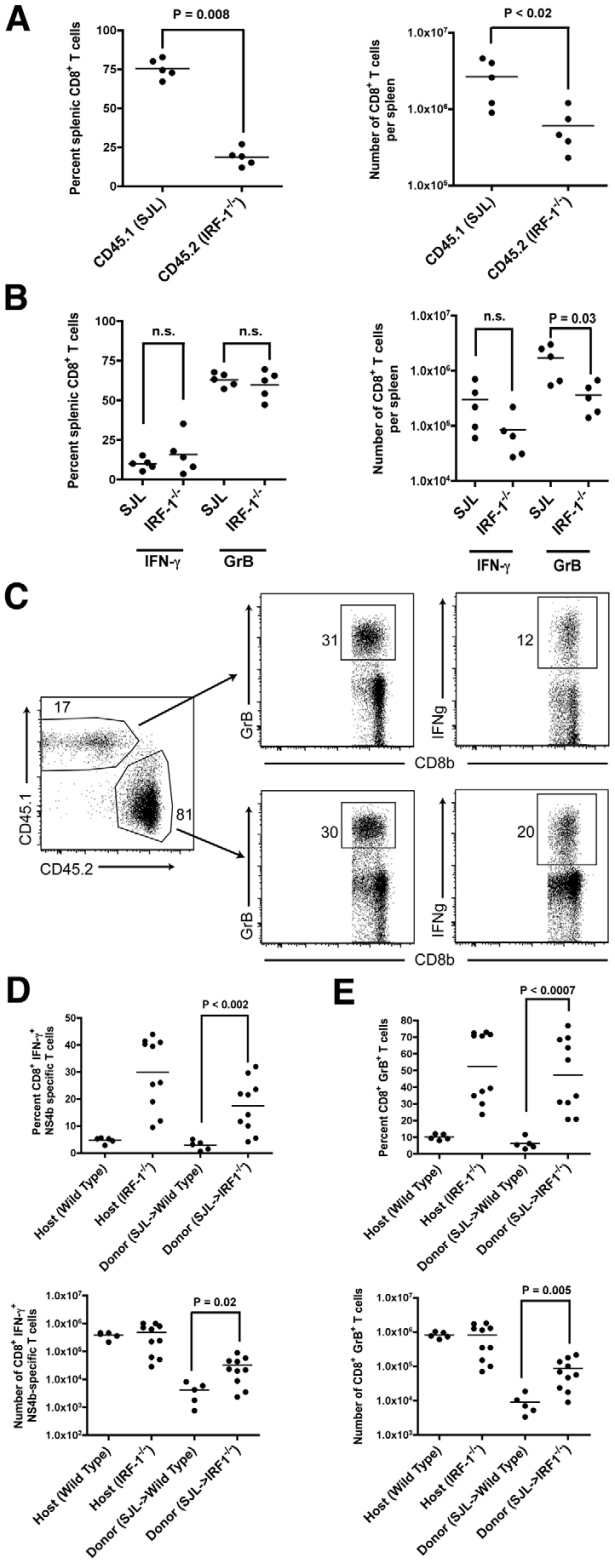
Adoptive transfer experiments identify cell-intrinsic and cell-extrinsic effects of IRF-1 on CD8^+^ T cell expansion. **A-B**. Equal numbers of naïve donor splenic CD8^+^ T cells from wild type SJL (CD45.1) or *IRF-1* ^-/-^ (CD45.2) mice were harvested, purified by negative selection, and transferred (2×10^6^ cells) into recipient *IRF-1* ^+/+^ *RAG1* ^-/-^ congenic mice. Mice were infected with WNV and eight days later, (**A**) splenocytes were harvested, and the number and percentage of CD45.1^+^ and CD45.2^+^ CD8^+^ T cells were enumerated. (**B**) Splenocytes were stimulated ex vivo with D^b^-restricted NS4B peptide, stained for CD3 and CD8b, and intracellularly for IFN-γ and GrB, and analyzed by flow cytometry. Results are the average of experiments with five mice. **C-E**. Naïve donor splenic CD8^+^ T cells from wild type SJL (CD45.1) mice were harvested, purified by negative selection, and transferred (1×10^6^ cells) into recipient CD45.2^+^ *IRF-1* ^-/-^ mice. Animals were infected with WNV and eight days later, splenocytes were harvested. (**C**) Flow cytometry dot plots showing percentage of donor (SJL (CD45.1)) and host (CD45.2^+^ *IRF-1* ^-/-^) CD8^+^ T cells that are positive for GrB or IFN-γ after ex vivo peptide restimulation. **D-E**. Graphical summary of the percentage and total number of IFN-γ^+^ or GrB^+^ SJL (CD45.1) CD8^+^ T cells after transfer into CD45.2^+^ *IRF-1* ^-/-^ mice and ex vivo peptide stimulation. A scatterplot of the data is shown and was generated from 5 wild type or *IRF-1* ^-/-^ mice. Note, the percentage and total number of IFN-γ^+^ or GrB^+^ wild type and *IRF-1* ^-/-^ CD8^+^ T cells in unperturbed yet WNV-infected mice are shown for comparison, some of which were performed in parallel. The bar indicates average values and asterisks denote differences that are statistically significant (P<0.05).

To assess how IRF-1 regulates the rapid expansion of CD8^+^ T cells after WNV infection, we performed competitive adoptive transfer experiments with wild type (CD45.1) and *IRF-1* ^-/-^ (CD45.2) CD8^+^ T cells into IRF-1^+/+^ *RAG1* ^-/-^ mice. Within 12 hours of transfer, slightly greater numbers (∼1.8-fold) of wild type (CD45.1) CD8^+^ T cells were observed in recipient *RAG1* ^-/-^ mice (data not shown). Animals subsequently were infected with WNV and harvested 8 days later for profiling of CD8^+^ T cells. Notably, we observed a cell-intrinsic effect of IRF-1 on the proliferative potential of CD8^+^ T cells as the wild type (CD45.1) cells rapidly expanded compared to *IRF-1* ^-/-^ (CD45.2) counterparts (4.1 and 4.4-fold increase in percentage and number, respectively, P≤0.02; **Fig 8A**). Consistent with this, although the percentage of NS4b-specific IFN-γ^+^ or GrB^+^ CD8^+^ T cells was equivalent (P > 0.6) in both the wild type (CD45.1) and *IRF-1* ^-/-^ (CD45.2) compartments (**Fig 8B**), the total number of *IRF-1* ^-/-^ (CD45.2) trended lower (3.5-fold lower for IFN-γ^+^ (P = 0.09) and 4.7-fold lower for GrB^+^ cells (P = 0.03)). Thus, CD8^+^ T cells lacking IRF-1 are at a competitive disadvantage compared to wild type cells within an *IRF-1^+/+^* stromal environment.

To determine whether an IRF-1 environment contributes to shaping the antigen-specific CD8^+^ T cell responses after WNV infection, we adoptively transferred wild type (CD45.1) CD8^+^ T cells into *IRF-1* ^-/-^ (CD45.2) or wild type (CD45.2) mice, and compared antigen-specific responses of the donor and host CD8^+^ T cells (**Fig 8C**). Remarkably, CD45.1 wild type CD8^+^ T cells in an *IRF-1* ^-/-^ mouse generated a larger WNV antigen-specific population compared to those observed in wild type mice (**Fig 8D and E**). These values approached but did not attain those observed with unperturbed, WNV-infected *IRF-1* ^-/-^ mice. These observations suggest that IRF-1 expression outside of the T cell compartment also regulates the magnitude of the antigen-specific CD8^+^ T cell response during WNV infection.

**Figure 9:**
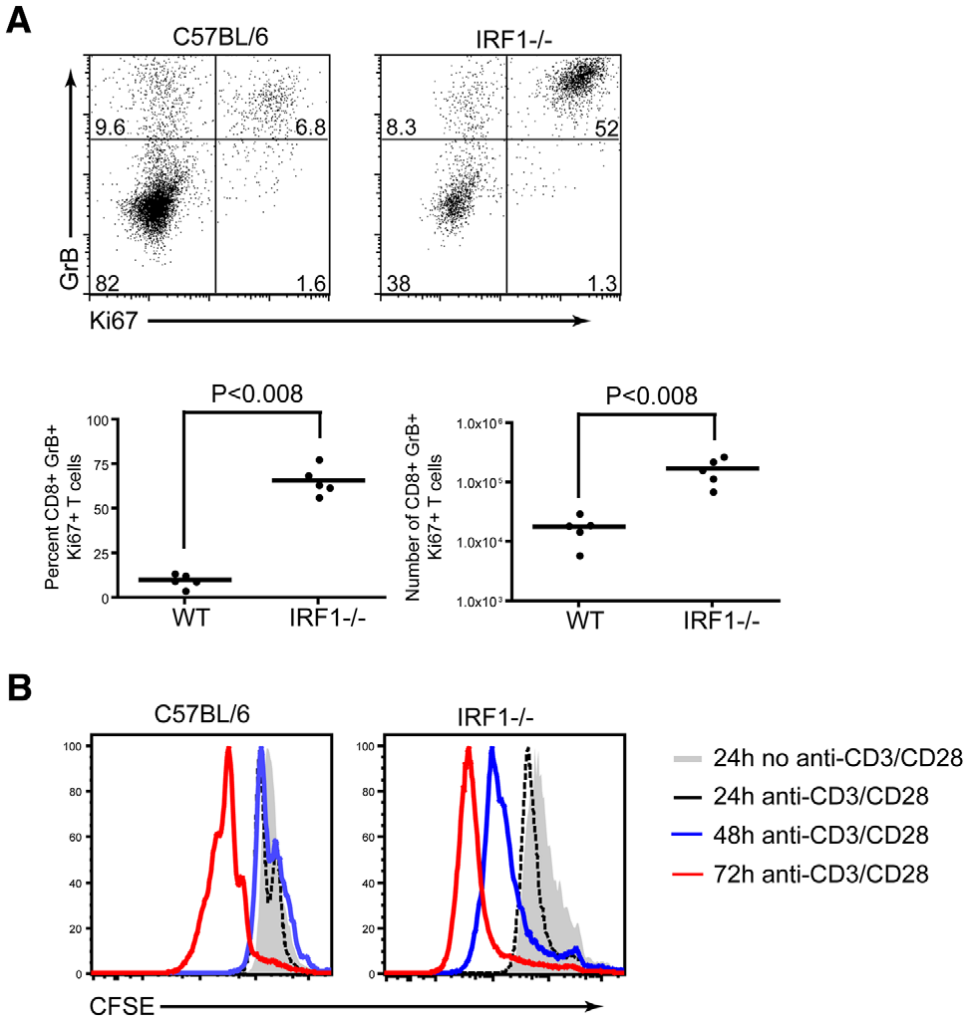
Proliferative capacity of *IRF-1* ^-/-^ CD8^+^ T cells. **A**. Assessment of CD8^+^ T cell proliferative capacity at day 8 after WNV infection. CD8^+^ T cells from wild type or *IRF-1* ^-/-^ mice were stained for CD8b, intracellular GrB and intranuclear Ki-67, and analyzed by flow cytometry. Representative flow cytometry dot plots gated on CD8b^+^ cells, showing the percentage of antigen-specific proliferating CD8b^+^GrB^+^Ki-67^+^ cells. Graphical summary of the percentage and total number of CD8b^+^GrB^+^Ki-67^+^ T cells. A scatterplot of the data is shown and was generated from two independent experiments with 5 wild type or *IRF-1* ^-/-^ mice. **B**. In vitro proliferative capacity of purified naïve CD8^+^ T cells. Representative flow cytometry profiles of carboxyfluorescein diacetate dilution from CD8b^+^ T cells stimulated with plate bound anti-CD3e (1 µM) and anti-CD28 (1 µM) at three time points. The data are representative of one individual mouse per group and the experiment was repeated three independent times with two mice per group each time.

To further understand the rapid expansion of antigen-specific CD8^+^ T cells in *IRF-1^-/-^* mice, we evaluated the percentage and number of cells that were proliferating at the peak of the response, based upon the expression of Ki67, a protein upregulated during the cell cycle. Ki67 expression was evaluated directly ex vivo, without peptide restimulation, in splenocytes of *IRF-1* ^-/-^ and wild type mice at 8 days after WNV infection. A significantly higher percentage (66 versus 10% P<0.008) and number (17×10^4^ versus 1.8×10^4^ cells, P<0.008) of WNV-specific CD8^+^ T cells in *IRF-1* ^-/-^ mice were proliferating at day 8 (**Fig 9A**). To address why there was an increase in proliferation of antigen-specific CD8^+^ T cells in *IRF-1* ^-/-^ mice during WNV infection, CD8^+^ T cells were isolated (∼90% purity) by negative selection, labeled with carboxyfluorescein diacetate, and stimulated with plate-bound anti-CD3ε and anti-CD28 in vitro. Notably, *IRF-1* ^-/-^ CD8^+^ T cells proliferated more rapidly than wild type cells at both 48 and 72 hours after stimulation (**Fig 9B**). Collectively, these results confirm that *IRF-1* ^-/-^ CD8 T cells have an intrinsic capacity to proliferate more rapidly in the context of stimulation through the T cell receptor, suggesting that IRF-1 acts to regulate T cell proliferation.

### Consequences of CD8^+^ T cell expansion in IRF-1^-/-^ mice

**Figure 10:**
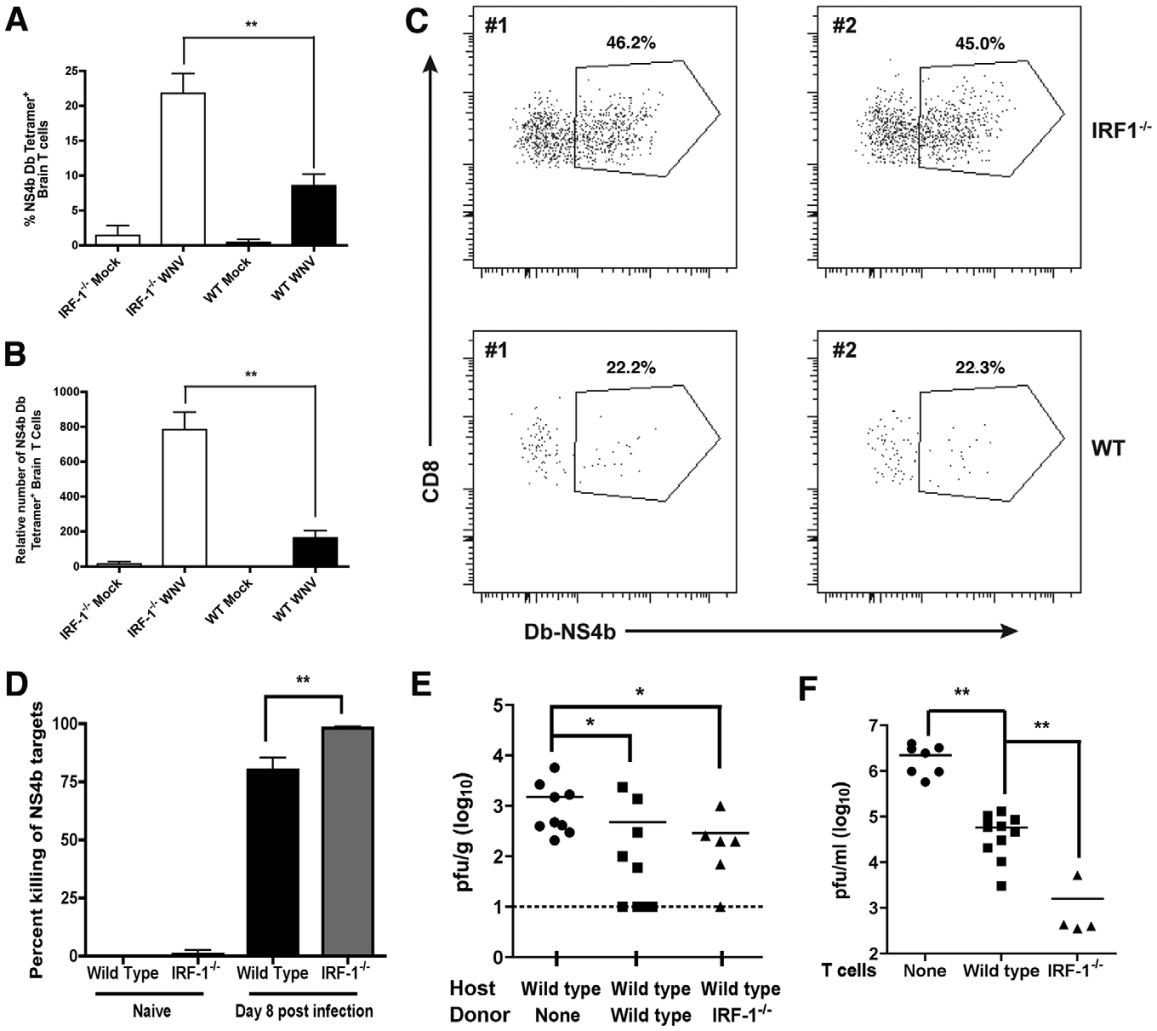
CD8^+^ T cell accumulation in the CNS of *IRF-1* ^-/-^ mice after WNV infection. Wild type and *IRF-1* ^-/-^ mice were inoculated with 10^2^ PFU of WNV by footpad injection, brains were harvested on day 8, and leukocytes were isolated after percoll gradient centrifugation. **A-B** The percentage and number of WNV-specific (Db-NS4b peptide tetramer^+^) CD8^+^ T cells was evaluated. The data are an average of experiments from 6 wild type and 5 *IRF-1* ^-/-^ mice. Asterisks indicate differences that are statistically significant. **C**. Representative flow cytometry profiles are shown of CD8 and WNV-specific tetramer staining of brain leukocytes from wild type and *IRF-1* ^-/-^ mice. **D**. In vivo cytolysis assay. At day 8 after infection, CFDA-labeled peptide-pulsed CD45.1 naive target cells (splenocytes from B6.SJL mice) were transferred to wild type or *IRF-1* ^-/-^ (CD45.2) WNV-infected or naïve mice (*n* = 6 to 12 per group from two independent experiments), and six hours later, mice were sacrificed and splenocytes analyzed for the ratio of peptide pulsed to non-pulsed cells by interrogating cells in the CD45.1 gate. The percentage of target cell killing in vivo was calculated by determining the ratio of peptide-pulsed versus unpulsed cells for each mouse, and by normalizing to that seen in naive mice. **E**. WNV titers from the brains (day 6 post infection) of wild type mice after adoptive transfer of wild type or *IRF-1* ^-/-^ WNV primed NS4b-specific CD8^+^ T cells (2×10^5^). CD8^+^ T cells were purified by negative selection and transferred 48 hours after infection. The data are an average of experiments from 6 to 9 recipient mice. Asterisks indicate differences that are statistically significant. **F**. CD8^+^ T cell-mediated control of WNV infection in cortical neurons. Cortical neurons from embryonic wild type mice were dissected and cultured. After infection with WNV at an MOI of 0.001 for one hour and extensive washing, positively-selected WNV-primed CD8^+^ T cells from wild type or *IRF-1* ^-/-^ mice were added at a 10∶1 E∶T ratio. Supernatants were harvested 48 hours later and the viral titer was measured by focus forming assay. Asterisks indicate differences that are statistically significant.

The accumulation of CD8^+^ T cells in the brain can be protective in the context of WNV infection depending on the numbers of migrating cells, the extent of neuronal infection, the timing of trafficking, and the inherent virulence of the viral strain [58], [60], [61], [64]. To assess whether the increase in relative numbers of WNV-specific CD8^+^ T cells in the spleen of *IRF-1* ^-/-^ mice was also observed in the CNS, we evaluated leukocyte accumulation in the brain. Leukocytes were recovered from the brains of wild type and *IRF-1* ^-/-^ mice at day 8 post-infection after perfusion. Equivalent percentages and numbers of CD45^high^/CD11b^high^ Mφ, CD45^low^/CD11b^high^ activated microglia, and CD4^+^ T cells were observed in the brains of WNV-infected wild type and *IRF-1* ^-/-^ mice (data not shown). In comparison, higher percentages (>2-fold) and total numbers (∼4-fold) of D^b^-restricted NS4b tetramer^+^ CD3^+^ CD8^+^ T cells were detected in the brains of *IRF-1* ^-/-^ mice (P<0.002) (**Fig 10A-C**). Thus, a deficiency of IRF-1 enhanced accumulation of antigen-specific CD8^+^ T cells in the brains of WNV-infected mice.

To determine the potential significance of the increased numbers of *IRF-1* ^-/-^ CD8^+^ T cells, we assessed their functional activity in vivo and ex vivo. When NS4b-peptide pulsed naïve splenocytes were adoptively transferred into WNV-infected wild type or *IRF-1* ^-/-^ mice, *IRF-1* ^-/-^ CD8^+^ T cells showed an equivalent if not slightly greater capacity to lyse target cells (98% versus 80%, P<0.03; **Fig 10D**). Importantly, NS4b-pulsed targets were not lysed when transferred into naïve wild type or *IRF-1* ^-/-^ mice. We next performed adoptive transfer studies with WNV-primed wild type or *IRF-1* ^-/-^ CD8^+^ T cells. To generate WNV-primed CD8^+^ T cells, wild type or *IRF-1* ^-/-^ mice were infected with WNV, and on day 7, spleens were harvested and 2×10^5^ NS4b tetramer^+^ CD8^+^ T cells were adoptively transferred into congenic five week-old wild type mice two days after WNV infection, and 4 days later (day 6 after infection), brains were harvested to evaluate the effect on control of WNV infection. While addition of naïve CD8^+^ T cells did not affect viral burden in the brain [60], wild type and *IRF-1* ^-/-^ CD8^+^ T cells both reduced viral titers (**Fig 10E**, P<0.02). To directly establish that *IRF-1* ^-/-^ CD8^+^ T cells could clear WNV from neurons, we used an ex vivo viral clearance assay with primary neurons derived from the cerebral cortex of wild type C57BL/6 mouse embryos [60]. One hour after infection, WNV-primed wild type or *IRF-1* ^-/-^ CD8^+^ T cells were added at an effector to target (E∶T) ratio of ten to one. At 48 hours after infection, the level of infectious WNV in the neuronal supernatants was measured by focus-forming assay. Addition of naïve CD8^+^ T cells does not reduce WNV infection in neurons [60]. In comparison WNV-primed CD8^+^ T cells significantly reduced (∼40 to 1400-fold, P<0.006) infectious virus production from infected cortical neurons, with an even greater (35-fold, P<0.02) inhibitory effect seen with IRF-1^-/-^ compared to wild type CD8^+^ T cells (**Fig 10F**). Collectively, these experiments suggest that WNV-primed *IRF-1* ^-/-^ CD8^+^ T cells are capable of controlling neuronal infection. However, this response is not sufficient to compensate for increased viral replication in peripheral tissues, which results in early and enhanced infection in the CNS of *IRF-1* ^-/-^ mice.

## Discussion

In this study, we identified IRF-1 as an essential regulator of the host immune response against WNV infection, and show that it governs processes of both the innate and adaptive immune responses that control outcome. *IRF-1* ^-/-^ mice were vulnerable to lethal infection with enhanced viremia, increased viral replication in peripheral tissues, altered tropism, and rapid dissemination into the CNS. Ex vivo analysis showed a cell-specific utilization of IRF-1 in controlling WNV replication. Although an absence of IRF-1 did not alter WNV infection in MEF, *IRF-1* ^-/-^ Mφ supported enhanced viral replication. Additionally, we identified a surprising phenotype with respect to the effects of IRF-1 on the induction of WNV-specific CD8^+^ T cells. Despite markedly fewer CD8^+^ T cells in naïve *IRF-1* ^-/-^ mice at baseline, we observed a rapid expansion in the periphery of antigen-specific CD8^+^ T cells that were IFN-γ^+^ and GrB^+^, which was associated with increased accumulation in the brain. Although *IRF-1* ^-/-^ CD8^+^ T cells expanded rapidly and were capable of killing targets and efficiently clearing virus from infected neurons, they did not attain sufficient numbers quickly enough to mitigate the increased infection in the CNS caused by the compromised early innate control of WNV in peripheral tissues.

Innate immunity and particularly type I (IFN-αβ), II (IFN-γ) and III (IFN-λ) IFN responses orchestrate control of infection by many DNA and RNA viruses [65], [66], [67]. Analogously, *IFN-αβR* ^-/-^ [15], *IFN-γ* ^-/-^ or *IFN-γR* ^-/-^ [68], [69], and *IFN-λR* ^-/-^ (IL28A) (H. M. Lazear and M. Diamond, unpublished results) mice all show enhanced susceptibility to WNV infection. The phenotype observed in the *IRF-1* ^-/-^ mice with WNV infection was in some ways similar to primary defects in IFN responses: increased replication in peripheral tissues at early time points that was associated with premature viral dissemination into the CNS. Because IRF-1 is important for efficient transduction of the antiviral signals downstream of IFN-γ [27], the early virologic phenotype in WNV-infected *IRF-1* ^-/-^ mice might be expected to phenocopy *IFN-γ* ^-/-^ mice [68], [69]. However, a comparison of the current data with published results revealed a more severe phenotype in *IRF-1* ^-/-^ mice, with higher and sustained infection in the spleen, altered tissue tropism with infection in the kidney, and higher titers in the brain and spinal cords between days 4 and 8. Thus, IFN-γ-independent immune regulatory functions must explain the greater susceptibility of *IRF-1* ^-/-^ compared to *IFN-γ* ^-/-^ mice.

IRF-1 is not required for induction of the IFN-αβ genes in MEF and Mφ in response to WNV infection, results that agree with experiments with NDV-infected fibroblasts [33], [40] or reovirus-infected myocytes [70]. Instead, IRF-3 and IRF-7 appear as the primary IRF transcriptional activators downstream of RIG-I and MDA5 recognition and IPS-1 signaling for efficient IFN-αβ gene induction after WNV infection of MEF and Mφ [18], [19]. The antiviral effect of IRF-1 was cell type-specific as enhanced WNV replication was observed preferentially in Mφ. An IRF-1-dependent antiviral signature is consistent with recent ectopic expression studies in human cells, which showed broad antiviral activity of IRF-1 against a range of viruses [25]. At present, we do not know the identity of the antiviral genes induced by IRF-1 that contribute directly to the virologic phenotype in *IRF-1* ^-/-^ Mφ. Comparative microarray analysis of WNV-infected wild type and *IRF-1* ^-/-^ MEF and Mφ is planned to define a set of uniquely induced genes that could function to limit WNV infection in a cell-type specific manner.

Previous studies have reported deficits in T cell function in *IRF-1* ^-/-^ mice including low levels of CD8^+^ T cells in peripheral lymphoid tissue [39], [40], [41]. Based on this, we expected a blunted and dysfunctional CD8^+^ T cell response after WNV infection in *IRF-1* ^-/-^ mice; as antigen-specific CD8^+^ T cells contribute to clearance of WNV-infected neurons [58], [59], [60], [61], [62], [71], we anticipated uncontrolled viral replication in the brain prior to death of the animals. Although we confirmed lower levels of peripheral CD8^+^ T cells in naïve *IRF-1* ^-/-^ mice, surprisingly, we observed rapid proliferation of antigen-specific CD8^+^ T cells such that a high fraction (∼30%) of CD8^+^ T cells in the spleen were directed against a single viral peptide epitope. Moreover, *IRF-1* ^-/-^ CD8^+^ T cells were fully capable of lysing target cells and clearing viral infection from neurons and the brain. This activity was consistent with a flattening of viral growth kinetics in the brain and spinal cord between day 6 and 8 in *IRF-1* ^-/-^ mice. In comparison, mice with targeted deletions in CD8^+^ T cells [58] or perforin [60] show logarithmic increases in CNS viral burden at this phase of infection.

How does IRF-1 regulate CD8^+^ T cell responses after WNV infection? Consistent with earlier studies, we observed a CD8^+^ T cell-intrinsic defect in proliferation and/or survival in vivo. This was most apparent in the increased ratio of wild type (CD45.1) to *IRF-1* ^-/-^ (CD45.2) CD8^+^ T cells within 12 hours of transfer to *RAG1* ^-/-^ mice. Additionally, by 8 days after infection, the ratio of total wild type (CD45.1) to *IRF-1* ^-/-^ (CD45.2) CD8^+^ T cells continued to increase. Given that the *RAG1* ^-/-^ mice are IRF-1 sufficient, this establishes a cell-intrinsic defect in CD8^+^ T cell expansion, and is consistent with bone marrow chimera reconstitution studies showing that *IRF-1* ^-/-^ thymocyte maturation was not restored in irradiated *IRF-1* ^+/+^ mice [39]. This phenotype could be due to altered responsiveness to IL-12, which has been observed in *IRF-1* ^-/-^ CD4^+^ T cells [43], [44], or possibly, other cytokines (e.g., IL-15 or IL-18) that regulate CD8^+^ T cell proliferation. Alternatively, IRF-1 signaling in antigen presenting or stromal cells may induce counter-regulatory networks or inhibit proliferation signals that result in tempered CD8^+^ T cell responses. Consistent with this possible function, we also observed blunted induction of CD4^+^CD25^+^FoxP3^+^ regulatory T cells in WNV-infected *IRF-1* ^-/-^ mice. A deficiency of regulatory T cells was previously shown to augment antigen-specific CD8^+^ T cell responses after WNV infection [63], although not to the extent observed in *IRF-1* ^-/-^ mice.

Within *IRF-1^-/-^* mice we observed an increase in the percentage and number of WNV-specific CD8^+^ T cells at the peak of infection due to increased proliferation. Our in vitro data supports this concept as *IRF-1* ^-/-^ CD8^+^ T cells proliferated to a greater extent than wild type cells after direct stimulation through the T cell receptor. Interestingly, adoptive transfer of wild type CD8^+^ T cells into an *IRF-1* ^-/-^ environment suggests that in vivo, a T cell extrinsic signal can drive the proliferation of antigen-specific cells. Historically, IRF-1 has been considered a negative regulator of cell proliferation, which in part explains its tumor suppressive activity (reviewed in [37]). At present, it remains uncertain as to the identity of the T cell-extrinsic signal that enhances the proliferation of antigen-specific cells in *IRF-1* ^-/-^ mice, although differential cell-type specific induction of key cytokines (e.g., IL-10 or IL-12) was not observed (**Fig S2**).

In summary, our experiments establish that IRF-1 has an essential function in the innate and adaptive immunity against WNV infection. In addition to its transduction of IFN-γ-dependent antiviral signals and functions in T cell lineage commitment in the thymus, IRF-1 appears to directly regulate antiviral genes in a cell-type specific and IFN-independent manner and be required for efficient expansion of regulatory T cells. Moreover, IRF-1 has both cell-intrinsic and -extrinsic functions in shaping CD8^+^ T cell responses, including stimulating both positive and negative regulatory networks depending on the cell type. Genetic profiling studies are planned with wild type and *IRF-1* ^-/-^ cells to identify novel gene signatures that can help define antiviral effector and immunomodulatory genes that inhibit viral infections and shape adaptive T cell responses.

## Materials and Methods

### Ethics statement

This study was carried out in strict accordance with the recommendations in the Guide for the Care and Use of Laboratory Animals of the National Institutes of Health. The protocol was approved by the Institutional Animal Care and Use Committee at the Washington University School of Medicine (Assurance Number: A3381-01). All inoculation and experimental manipulation was performed under anesthesia that was induced and maintained with ketamine hydrochloride and xylazine, and all efforts were made to minimize suffering.

### Viruses

The WNV strain (3000.0259) was isolated in New York in 2000 [72] and passaged once in C6/36 *Aedes albopictus* cells to generate a stock virus that was used in all experiments.

### Mouse experiments

Wild type and congenic *RAG1* ^-/-^ C57BL/6 mice were obtained commercially (Jackson Laboratories). C57BL/6.SJL-Ptprc^a^/BoyAiTac mice were purchased (Taconic) and are congenic with respect to C57BL/6 mice except at the Ly5.1 (CD45.1) locus. *IRF-1* ^-/-^ mice were originally generated by T. Taniguchi [3], [34], [73] and obtained on a C57BL/6 background (kindly provided by T. Taniguchi and K. Fitzgerald). All mice were genotyped and bred in the animal facilities of the Washington University School of Medicine under pathogen free conditions, and experiments were performed in strict compliance with Washington University Animal Studies guidelines. Eight to twelve week old mice were used for all in vivo studies. For peripheral infection, 10^2^ PFU of WNV was diluted in Hanks balanced salt solution (HBSS) supplemented with 1% heat-inactivated fetal bovine serum (FBS) and inoculated by footpad injection in a volume of 50 µl.

### Quantification of tissue viral burden and viremia

To monitor viral spread in vivo, mice were infected with 10^2^ PFU of WNV by footpad inoculation and sacrificed at days 1, 2, 4, 6 and 8 after inoculation. After cardiac perfusion with PBS, organs were harvested, weighed, homogenized and virus was titrated by standard plaque assay as described [57]. Viremia was measured by analyzing WNV RNA levels using fluorogenic quantitative RT-PCR (qRT-PCR) as described [15].

### Primary cell culture and viral infection

#### (a) Macrophages

Bone marrow derived Mφ were generated according to published protocols [6]. Briefly, bone marrow cells were isolated from mice and cultured for seven days in the presence of macrophage colony-stimulating factor (M-CSF) (PeproTech) to generate Mφ. Multi-step viral growth curves were performed after infection at a multiplicity of infection (MOI) of 0.01 for Mφ. Supernatants were titrated by plaque assay on BHK21 cells. To test for induction of IFN-α and β genes after WNV infection, 5×10^5^ Mφ were infected at an MOI of 0.1 and IFN-α and β mRNA was measured by qRT-PCR.

#### (b) Fibroblasts

Mouse embryo fibroblasts were generated from wild type and *IRF-1* ^-/-^ 14-day-old embryos and maintained in DMEM supplemented with 10% FBS. Cells were used between passages 2 and 4 for all experiments. Multi-step virus growth curves were performed after infection at an MOI of 0.001.

### Quantification of IFN-α and β mRNA by qRT-PCR

Total RNA was isolated from lymph nodes or primary cells by using the RNeasy kit according to the manufacturer's instructions (Qiagen). During the isolation, to remove any contaminating DNA, samples were treated with RNAse-free DNAse (Qiagen). IFN-α and β mRNA were amplified and quantified from total RNA by qRT-PCR as previously described [47]. The following primers and probes were used to amplify murine IFN-α and IFN-β mRNA: IFN-α, forward primer, 5′-CTTCCACAGGATCACTGTGTACCT-3′, reverse primer, 5′TTCTGCTCTGACCACCTCCC3′, probe, 5′-FAM-AGAGAGAAGAAACACAGCCCCTGTGCC-TAMRA-3′; IFN-β, forward primer, 5′-CTGGAGCAGCTGAATGGAAAG-3′, reverse primer, 5′-CTTCTCCGTCATCTCCATAGGG-3′, probe 5′-FAM-CAACCTCACCTACAGGGCGGACTTCAAG-TAMRA-3′. To analyze the relative fold induction of IFN-α and IFN-β mRNA, 18S rRNA expression levels were also determined for normalization by using the Ct method as described [47].

### Measurement of IFN activity by bioassay

Levels of biologically active IFN in serum were measured using an EMCV L929 cytopathic effect bioassay as described previously [6]. Results were compared with a standard curve using recombinant mouse IFN-α (PBL Laboratories).

### IFN treatment and neutralization assays

For IFN inhibition assays, wild type or *IRF-1* ^-/-^ Mφ were pretreated with increasing doses of IFN-β or IFN-γ (PBL Laboratories) for 24 hours and then infected with WNV at an MOI of 0.1. Supernatants were harvested 48 hours after infection and titered by plaque assay. For the IFN-γ neutralization assay, various concentrations of exogenous IFN-γ were mixed with 20 µg of a hamster anti-mouse IFN-γ neutralizing MAb (H22) [55] or isotype control for 1 hour at 37°C. Alternatively, wild type Mφ were pretreated with the IFN-γ with or without MAbs mixture for 24 hours and then infected with WNV at an MOI of 0.1. Supernatants were harvested 48 hours post infection and titered by plaque assay. To test whether H22 (and neutralization of IFN-γ) affected viral replication, multi-step growth curves with wild type or *IRF-1* ^-/-^ Mφ were performed in the presence of 20 µg of H22 antibody or isotype control after infection at an MOI of 0.01.

### WNV-specific antibody and CD8^+^ T cell responses

The levels of WNV-specific IgM and IgG were determined using an ELISA against purified WNV E protein [74]. The focus reduction neutralization assay was performed as described previously [75]. Intracellular IFN-γ or TNF-α staining was performed on splenocytes using a previously identified D^b^-restricted NS4B peptide or K^b^-restricted E peptide in a re-stimulation assay with 1 µM of peptide and 5 µg/ml of brefeldin A (Sigma) as described [71]. The number and percentage of CD4^+^CD25^+^FoxP3^+^ regulatory T cells in the spleen of naïve or WNV-infected wild type or *IRF-1* ^-/-^ mice was measured using the regulatory T cell staining kit (Ebioscience). Granzyme B (Invitrogen, clone gb12) intracellular staining was performed without stimulation using the staining solutions for FoxP3. Intranuclear staining for Ki67 antigen (BD Pharmingen, clone B56) was performed on CD8^+^ T cells ex vivo without additional stimulation using the FoxP3 staining solutions. In some experiments WNV-specific CD8^+^ T cells were stained with a D^b^-restricted NS4B peptide tetramer (NIH Tetramer Core Facility, Emory University, Atlanta, GA). Samples were processed by multi-color flow cytometry on an LSR or FACSCalibur flow cytometer (Becton Dickinson) and analyzed with FlowJo software (Treestar).

### Adoptive transfer experiments

Equal numbers of naïve donor splenic CD8^+^ T cells from wild type (CD45.1) or *IRF-1* ^-/-^ (CD45.2) mice were harvested, purified by negative selection using antibody-coated magnetic beads (anti-NK1.1, anti-B220, anti-CD4, and anti-MHC class II; Miltenyi Biotec), and transferred (2×10^6^ total cells) into recipient *RAG1* ^-/-^ mice. One day later mice were bled to confirm the reconstitution, and then infected with WNV (10^2^ PFU via a subcutaneous route). Eight days later, splenocytes were harvested, and analyzed for activation and cytokine expression as described above. A similar procedure was followed for the adoptive transfer of CD8^+^ T cells (2×10^6^ total cells) from wild type CD45.1 SJL donors into recipient wild type (CD45.2) or *IRF-1* ^-/-^ (CD45.2) hosts. Purity of transferred T cell populations, which ranged from 88 to 92% CD8b^+^, was evaluated by flow cytometry.

To evaluate the ability of activated CD8^+^ T cells to reduce viral burden, wild type and *IRF-1* ^-/-^ CD8^+^ T cells were isolated using antibody-coated magnetic beads at day 7 after infection and 2×10^5^ NS4b tetramer^+^ cells were adoptively transferred by intravenous route 48 hours after subcutaneous WNV (10^2^ PFU) infection of 5 week-old wild type mice. Mice were euthanized at day 6 after infection for analysis of viral burden in the brain by focus forming assay.

### CD3^+^ T cell stimulation

Naïve splenic CD8a^+^ T cells were purified from wild type and *IRF-1* ^-/-^ mice by positive selection using antibody-coated magnetic beads (anti-CD8a, Miltenyi Biotec). After purification, CD8^+^ T cells were washed twice with PBS and incubated with 1 µM carboxyfluorescein diacetate succinimidyl ester in PBS for 10 minutes at 37°C. The reaction was quenched by adding an equal volume of RPMI 1640 supplemented with 10% FBS, 0.1 mM β-mercaptoethanol, penicillin, and streptomycin. After washing, purified labeled CD8^+^ T cells (10^6^) were plated in individual flat bottom 96 well tissue culture treated plates in the RPMI medium described above, in the presence or absence of 1 µM anti-CD3ε (Biolegend, clone 2c11 NA/LE) and 10 µM anti-CD28 (BD Biosciences, clone 37.51). Cells were harvested at 24, 48 and 72 hours, washed twice with PBS, and analyzed by flow cytometry.

### Leukocyte isolation from CNS

Quantification of infiltrating CNS lymphocytes was performed as previously described [76]. Briefly, wild type and *IRF-1* ^-/-^ mouse brains were harvested on day 8 after infection, dispersed into single cell suspension with a cell strainer and digested with 0.05% collagenase D, 0.1 µg/ml trypsin inhibitor TLCK, 10 µg/ml DNase I and 10 mM of HEPES (Life Technologies) in HBSS for 1 hour. Cells were separated by discontinuous Percoll-gradient (70/37/30%) centrifugation for 30 min (850 x g at 4°C). Cells were then counted and stained for CD4, CD8, CD45 and CD11b with directly conjugated antibodies (BD Pharmingen) for 30 minutes at 4°C, and then fixed with 1% paraformaldehyde. WNV-specific CD8^+^ T cells were identified using a D^b^-restricted NS4B peptide tetramer.

### In vivo CD8^+^ T cell cytolysis assay

In vivo killing of target cells was performed as previously described [77]. Briefly, splenocytes from B6.SJL (CD45.1) mice were isolated. Half of the cells were labeled with carboxyfluorescein diacetate succinimidyl ester (CFDA) at 500 nM and the remainder was labeled with 5 nM CFDA. After labeling, cells labeled with 500 nM CFDA were pulsed for one hour at 37°C with 1 µM NS4B 2488-2496 peptide, whereas the 5 nM CFDA cells were not pulsed with peptide. Both sets of cells were counted and equal numbers were mixed and injected intravenously (10^7^ cells total per mouse) into recipient WNV-infected (at day 8 after infection) wild type or *IRF-1* ^-/-^ mice. After 8 hours, the mice were sacrificed and splenocytes were gated on CD45.1 cells (donor cells). The percent killing of target cells was calculated: (1 – (ratio immune/ratio naive)) ×100. Ratio equals the number of NS4B peptide-coated targets/number of reference targets [77].

### Addition of CD8^+^ T cells to WNV-infected neurons

Purified CD8^+^ T cells were incubated with WNV-infected cortical neurons as described previously [6]. Briefly, cortical neurons were isolated and plated onto 24 well plates at 3×10^5^ neurons per well. Four to five days after plating, neurons were infected with WNV at an MOI of 0.001 for one hour. Neurons were washed thrice with warm media and 2×10^6^ (effector∶target ratio of 10∶1) CD8^+^ T cells, isolated by positive selection from wild type or *IRF-1* ^-/-^ splenocytes at eight days post infection, were added. Individual wells were harvested at 48 hours after infection, and WNV titers were measured by focus-forming assay.

### Statistical analysis

For in vitro experiments, an unpaired T-test was used to determine statistically significant differences. For viral burden and T cell analysis, differences were analyzed by the Mann-Whitney test. Kaplan-Meier survival curves were analyzed by the log rank test. All data were analyzed using Prism software (GraphPad Prism4, San Diego, CA).

### Supplemental methods

Additional materials and methods are provided (**Text S1**).

## Supporting Information

Figure S1 CD8^+^ NS4b^+^ cells express GrB from naïve and WNV-infected mice.
(TIF)

Figure S2 Serum cytokines from infected and uninfected wild type and *IRF-1* ^-/-^ mice.
(TIF)

Text S1 Supplemental methods.
(DOC)
