# Supplementary figures and images for: Interferon Regulatory Factor-1 (IRF-1) Shapes Both Innate and CD8+ T Cell Immune Responses against West Nile Virus Infection
Source: PLoS Pathog. 2011 Sep 1;7(9):e1002230. doi: 10.1371/journal.ppat.1002230 (PMC3164650; doi:10.1371/journal.ppat.1002230)

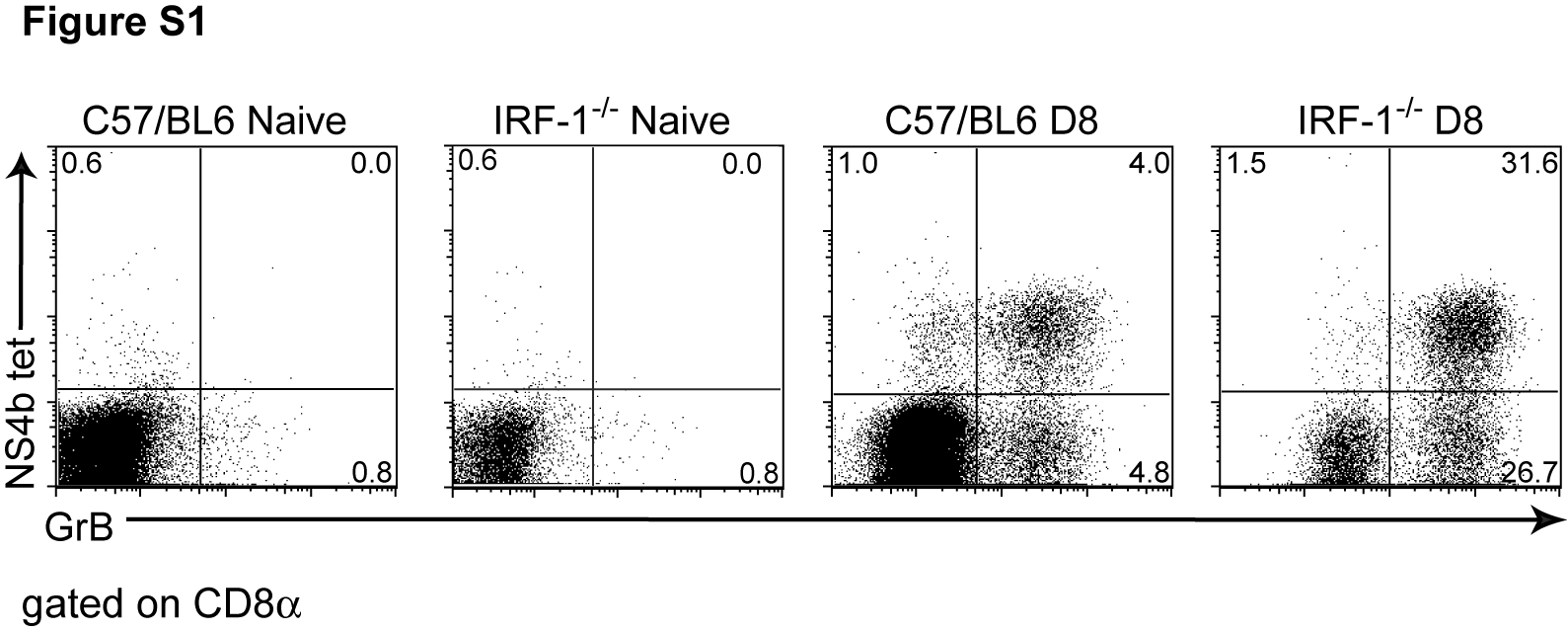

Supplement: Figure S1 — CD8+ NS4b+ cells express GrB from naïve and WNV-infected mice. (TIF) [file ppat.1002230.s001.tif]

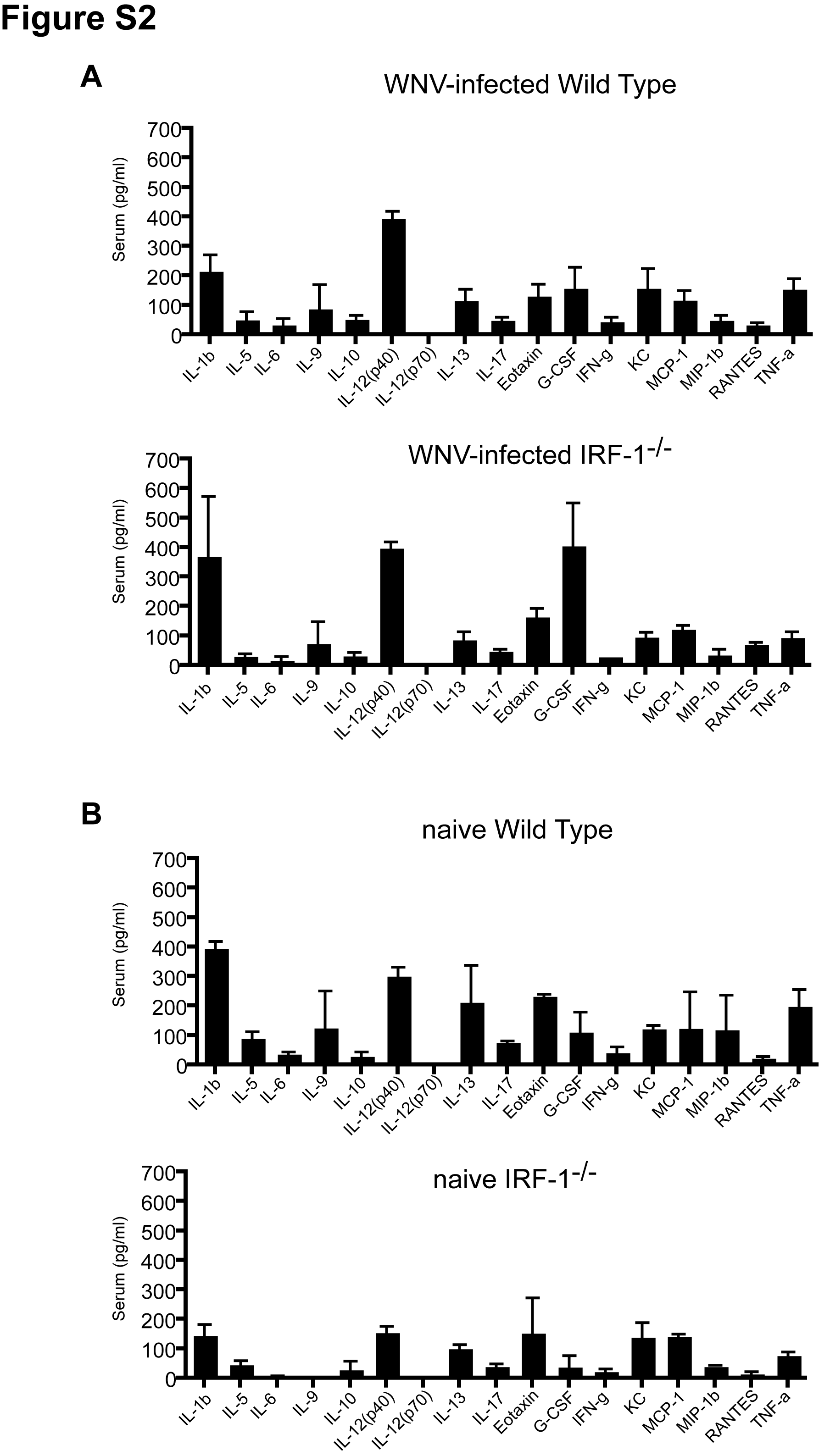

Supplement: Figure S2 — Serum cytokines from infected and uninfected wild type and IRF-1 -/- mice. (TIF) [file ppat.1002230.s002.tif]
