# Supplementary material for: Interferon Regulatory Factor-1 (IRF-1) Shapes Both Innate and CD8+ T Cell Immune Responses against West Nile Virus Infection
Source: PLoS Pathog. 2011 Sep 1;7(9):e1002230. doi: 10.1371/journal.ppat.1002230 (PMC3164650; doi:10.1371/journal.ppat.1002230)
Supplement: Text S1 — Supplemental methods. (DOC) [file ppat.1002230.s003.doc]

**SUPPORTING INFORMATION**

**Figure S1**. Representative flow cytometry dot plots showing percentage of CD8^+^ NS4b^+^ cells that express GrB from naïve and WNV-infected (at day 8) wild type and *IRF-1*^-/-^ mice. Flow profiles are gated on live CD3^+^ CD8α^+^ cells.

**Figure S2**. Cytokine bioplex assay using serum from (**A**) infected and (**B**) uninfected wild type and *IRF-1*^-/-^ mice. Twenty-three cytokines were evaluated from serum, IL-1α, IL-2, IL-3, IL-4, GM-CSF, and MIP-1α were below the limit of detection in all experimental samples and are not shown. This experiment was performed with serum from *n* = 4 infected mice and *n* = 2 naïve mice.

**TEXT S1**

***Cytokine bioplex assay*.** The cytokine bioplex assay was performed on serum samples from both naïve mice and WNV-infected (day 8) wild type and *IRF-1*^-/-^ mice. The BioPlex Pro Assay was performed according to the manufacturer’s protocol (BioRad). The cytokine screen included IL-1α, IL-1β, IL-2, IL-3, L-4, IL-5, IL-6, IL-9, IL-10, IL-12p40, IL-12p70, IL-13, IL-17, Eotaxin, G-CSF, GM-CSF, KC, MCP-1, RANTES, MIP-1α, MIP-1β, IFN-γ, and TNF-α.
